# Commercial Silver-Based Dressings: In Vitro and Clinical Studies in Treatment of Chronic and Burn Wounds

**DOI:** 10.3390/antibiotics13090910

**Published:** 2024-09-23

**Authors:** Sweta Shrestha, Bo Wang, Prabir K. Dutta

**Affiliations:** 1 ZeoVation Inc., Columbus, OH 43212, USA; sweta.shrestha@zeovation.com (S.S.); bowang@zeovation.com (B.W.); 2 Department of Chemistry and Biochemistry, The Ohio State University, Columbus, OH 43210, USA

**Keywords:** biofilms, wound healing, wound care, silver toxicity, dressing matrix

## Abstract

Chronic wounds are a major health problem because of delayed healing, causing hardships for the patient. The infection present in these wounds plays a role in delayed wound healing. Silver wound dressings have been used for decades, beginning in the 1960s with silver sulfadiazine for infection prevention for burn wounds. Since that time, there has been a large number of commercial silver dressings that have obtained FDA clearance. In this review, we examine the literature involving in vitro and in vivo (both animal and human clinical) studies with commercial silver dressings and attempt to glean the important characteristics of these dressings in treating infected wounds. The primary presentation of the literature is in the form of detailed tables. The narrative part of the review focuses on the different types of silver dressings, including the supporting matrix, the release characteristics of the silver into the surroundings, and their toxicity. Though there are many clinical studies of chronic and burn wounds using silver dressings that we discuss, it is difficult to compare the performances of the dressings directly because of the differences in the study protocols. We conclude that silver dressings can assist in wound healing, although it is difficult to provide general treatment guidelines. From a wound dressing point of view, future studies will need to focus on new delivery systems for silver, as well as the type of matrix in which the silver is deposited. Clearly, adding other actives to enhance the antimicrobial activity, including the disruption of mature biofilms is of interest. From a clinical point of view, the focus needs to be on the wound healing characteristics, and thus randomized control trials will provide more confidence in the results. The application of different wound dressings for specific wounds needs to be clarified, along with the application protocols. It is most likely that no single silver-based dressing can be used for all wounds.

## 1. Introduction

Chronic wounds, i.e., non-healing wounds, are a major health problem. Examples of chronic wounds are vascular wounds, diabetic foot, and pressure ulcers [1,2,3]. More than 6 million people in the United States suffer from ulcers, and this problem is particularly acute amongst the elderly [2,4]. Cases of diabetes are also increasing and, by 2030, these numbers will exceed 20 million, and 15% of these cases will develop diabetic foot ulcers [3,4,5,6,7]. Chronic wounds are characterized clinically by increasing pain in the wound area, along with bad odor, wound breakdown, and friable granulation tissue, and taking longer than 3 months to achieve anatomical integrity [2,3,5,6,7]. The reason for the delayed healing is that the normal phases of wound healing are disrupted in chronic wounds, infection is manifested by the presence of biofilms, and prolonged inflammatory response causes tissue damage. It is estimated that $96.8B is spent on wound care in the US, with about $7.2B for chronic wound care [8]. In 2014, it was estimated that 15% of Medicare patients had wound infections and 4% had surgical site infections [9]. Three million people have hard-to-heal pressure ulcers, which take months to years to completely heal, and costs for treating pressure ulcers are $26.8B annually [10]. Another class of wounds that can get infected are burns, which are acute wounds. Chemical, thermal, electrical, and radioactive exposures can cause burns [11]. Burn wounds lead to tissue necrosis and secondary infections [11].

Wound healing is a complex process involving hemostasis, inflammation, granulation, epithelization, contraction, and ending with remodeling [2,4,12]. Inflammation in the early stages prevents microorganisms and reduces necrosis [13]. Increased fibroblasts aid in the synthesis of collagen, elastin, glycoproteins, and proteoglycans, thereby promoting wound closure [14]. Inappropriate external and physiological interventions can disrupt this pathway, compromising the healing process [12,13]. For example, if inflammation is prolonged, matrix metalloproteases and serine proteases secreted from the fibroblasts can impair healing [15]. Major physiological changes in the wound include infection, altered blood flow, and hypoxia which influences phagocytosis, cellular failure and trauma, increased inflammation.

**Figure 1:**
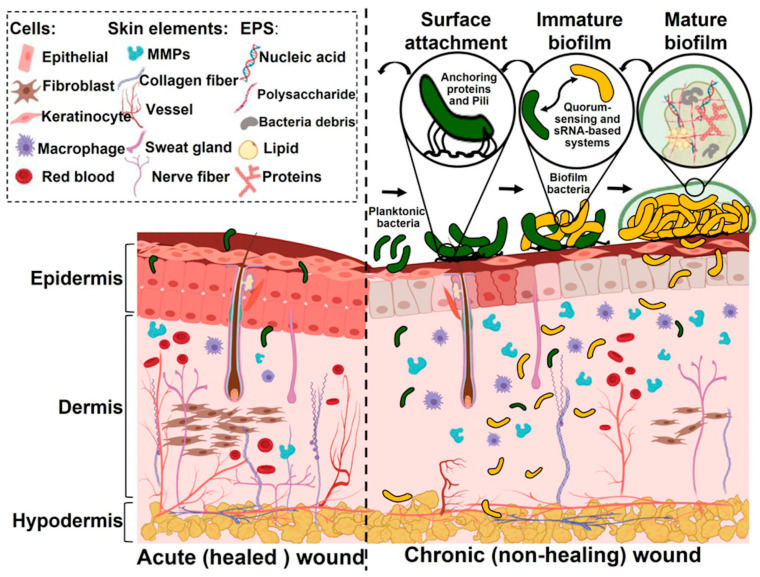
Contrast between an acute wound and a biofilm-infected chronic wound (Taken with permission from reference [19]).

Infections can play a major role in thwarting the healing process in chronic and burn wounds. Wounds are heterogeneous, with slough, exudate, and necrotic tissue, all sites for bacteria and biofilm development [16]. Bacterial colonization of the wound can lead to the production of toxins, alkaline pH (7.3–8.9), and lower tissue oxygen levels and neutrophil activation [17,18]. Most infections are polymicrobial, containing both aerobic and anaerobic bacteria, and the larger the number of pathogens, the infection will increase. Figure 1 contrasts the wound healing process between an acute wound and a chronic biofilm-infected wound [19].

In this review, we focus on infected wounds treated with commercial silver-based dressing [4,11,20,21,22,23,24,25,26,27,28]. The goal of this review is to provide the reader with the potential of silver-based dressings in treating wounds. We have focused only on commercial dressings and, though it is difficult to predict which dressings are most appropriate for a specific application, this review will provide some sense of the advantages and disadvantages of the dressings, based on in vitro and in vivo studies. Table 1, Table 2 and Table 3 summarize the in vitro/in vivo studies of silver-based dressings. For the clinical studies, we have separated them into chronic and burn wounds (Table 2 and Table 3, respectively). By presenting most of the information in a systematic tabular form, it is relatively easy for the reader to find detailed characteristics of a dressing as well as clinical information on a particular dressing. Usually, in most review articles, the tables are abbreviated, and the reader is directed towards the original reference. By providing more detailed information in the tables, we believe that the review will be more useful to researchers. It is difficult to compare the clinical performance of the different dressings, considering that the methodology of clinical studies varies considerably. The narrative part of the review focuses on the important features of silver-based dressings, their physical characteristics, and the relevant structural features that explain the physiological activity of the dressings. General conclusions are drawn from clinical studies. There are several reasons why such a review is necessary. First, this review will be useful in designing the next generation of silver-based wound dressings, based on the limitations of the current dressings. Second, it provides the reader with the current scope of commercial dressings that are available, as well as applications of many of these dressings in a clinical setting. Third, the variety of endpoints in applications of these wound dressings in clinical applications is highlighted.

## 2. Methods Section

The methodology for writing this review was as follows. In order to identify the commercial dressings, we carried out searches on the web using silver dressings as the keyword. Information about these dressings was obtained from the websites of the companies. This information was double-checked in some cases from the publications that used these dressings. For the in vitro and in vivo studies, searches were carried out using the keyword ‘silver wound dressing’ in SciFinder, PubMed, and Scopus. The years that we focused on were primarily from the years 2000 and beyond, although we also went back to some original references before the year 2000. Papers that did not explicitly use silver in the wound dressing were excluded. For papers that used silver dressing, we did not make any critical judgments to remove them from consideration. There are numerous papers on silver as an antimicrobial, but since the focus of this article is on wound dressings, we have used those references that provide information on the activity of silver wound dressings.

## 3. Bacterial Infection and Biofilms

Bacteria’s self-defense mechanism in a natural environment is to create three-dimensional structures referred to as biofilms, in which the bacterial colonies are enclosed by a self-generated extracellular polymeric substance (EPS) matrix that protects the bacteria [16,17,80]. Biofilms attached to surfaces harbor more bacteria than what is in the surroundings, e.g., in a slime layer rock in a Canadian alpine stream, the amount of bacteria in the biofilm exceeded the planktonic bacteria by a factor of 1000–10,000 [81,82]. Biofilms are ubiquitous and impact human and animal health, agriculture, food processing, wastewater treatment, and marine infrastructure. The costs to the economy due to biofilms are estimated to be $5T globally [83]. Biofilms can appear on catheters, prosthetic joints, cardiac valves, and implants, and are estimated to cause $1.6B in expenses [8,83].

The EPS matrix is mostly water (97%) and contains, in decreasing order, polysaccharides, lipo-associated teichoic acids, and cellulose, followed by proteins and extracellular DNA, and ions. EPS layer thickness can range from tens of microns to hundreds of microns, with varying morphology, including flat, fluffy, filamentous structures, along with pores and channels for nutrient transport. The EPS enclosure promotes cell-to-cell contact, which promotes bacterial genetic alterations. Biofilms are diverse, containing polymicrobial colonies, with phenotypes referred to as persister cells [84] that have a high antimicrobial tolerance as well as small colony variants effective at forming new biofilms [16,17]. In the polymicrobial biofilms, the interaction of the bacteria promotes survival [1]. The presence of the EPS matrix also leads to overexpression of stress-responsive genes and altered oxygen gradients [85]. Bacteria trapped within the biofilm cannot be reached by phagocytic neutrophils and macrophages [19]. The immune system’s extended fight with biofilms can cause damage to the host tissue [18]. Antimicrobial agents that are active against planktonic bacteria are not effective in killing the EPS-enclosed bacteria [17]. Systematic antibiotic therapy is not useful for biofilm-infected chronic wounds [86]. Diverse microflora and multispecies biofilm formation are reasons that wounds become hard to treat by antibiotic therapy [85,86].

The clinical definition of bacterial infection is dependent on the bacterial population, with the level of >10^5^ bacteria (CFU/mm^3^ of tissue) being considered as infective [87]. Twenty-eight bacterial species were identified in wound swab samples from 213 patients with different types of wounds, the most common being *Staphylococcus aureus* (*S. aureus*), *Pseudomonas aeruginosa* (*P. aeruginosa*), *Proteus mirabilis*, *Escherichia coli* (*E. coli*), and *Corynebacterium* spp. [88]. Chronic venous leg ulcers were found to contain *S. aureus* (93.5% of the investigated ulcers), *Enterococcus faecalis* (71.7%), *P. aeruginosa* (52.2%), coagulase-negative *Staphylococci* (45.7%), proteus species (41.3%), and anaerobic bacteria (39.1%) [89]. The distribution of bacteria in polymicrobial wounds is not uniform, e.g., *P. aeruginosa* occurs deeper in wounds (50–60 μm), whereas *S. aureus* was found more on the surface of the wound (20–30 μm) [1,89,90].

Immunocompromised humans are ideal hosts for biofilms, providing the appropriate nutrients, humidity, and temperature for the biofilms to thrive [19]. Biofilm formation is evident in diseases, such as cystic fibrosis, osteomyelitis, conjunctivitis, vaginitis, urethritis, endocarditis, pediatric respiratory infections, and oral diseases [17]. NIH estimates that 80% of microbial infections contain biofilms [17]. Biofilms are associated with 78.2% of chronic wounds and 6% of acute infections. For hospital-acquired infections, 1.7M were associated with biofilms [19].

Biofilm formation in wounds is a dynamic process, and a mature biofilm can develop in 24 h [19]. There are many reports of the presence of biofilms in chronic wounds [89,90,91,92]. In an electron microscopy study, 30 out of 50 chronic wound specimens from human subjects were found to contain biofilms, whereas only 1 of the 16 acute wound specimens from human subjects had biofilms [92]. *S. aureus* and *P. aeruginosa* were found in human chronic wound samples, with the latter penetrating deeper into the wounds [89]. The presence of polymicrobial biofilms impedes the healing process and increases the costs of wound care [93,94]. The wound bed is also ripe for providing nutrients via exudates, and the necrotic tissues can act as sites for biofilm attachment [95]. Biofilms lead to low-grade and persistent inflammation and slow down epithelization and granulation tissue formation, which are critical to wound healing [1,91]. Biofilms also impair the host immune response [95]. Clinically, biofilms in wounds are detected by the presence of yellow exudate and necrotic tissue [19]. However, the presence of biofilms in wounds is not without controversy, with at least one analysis stating that in vivo proof is not conclusive, primarily because no established method for the detection of biofilms in a clinical setting is available [96].

Biofilms are difficult to eradicate [1]. Wounds infected by bacteria and bacterial biofilms take longer to heal [95,97,98]. The EPS layer in biofilms in chronic wounds is structurally robust and behaves like viscoelastic solids, requiring mechanical disruption for access to the entrapped bacteria [16,99]. Ultrasound debridement is also possible [19]. It is also possible to target the constituents of the EPS layer, including the eDNA, polysaccharides, and proteinaceous adhesins, and this is an area of active research [16]. Other strategies for biofilm disruption include photodynamic therapy and electrically generated peroxides [16] and chelating agents, e.g., ethylene diamine tetra acetic acid (EDTA) [19]. Though mechanical debridement is effective, it can cause damage to healthy tissues, pain, and the spread of bacteria [19,99].

Typical treatment of chronic wounds (BBWC—biofilm-based wound care) involves removing the debris and eschar with saline/wound cleaners (which contain surfactants), mechanical debridement, and treatment with topical antimicrobials and or antimicrobial wound dressings to kill the pathogenic bacteria set loose (planktonic) by debridement [99]. The bacteria released during debridement needs to be killed since biofilms can form again in hours to days [91]. Debridement alone can decrease bacteria by one to two log_10_, which is not sufficient to impede bacterial regrowth [34,100]. It is unclear if antimicrobial wound dressings can have an impact on wound healing without wound debridement [80].

## 4. Wound Dressings

The purpose of using wound dressings is to promote wound healing. However, because of the complexity of wound healing, a single wound dressing may not be appropriate for all types of wounds. Thus, many wound management strategies are being developed [101]. Sometimes, a healed wound cannot be determined by visual observation as the skin barrier function in a visually healed wound may not be functioning properly [80]. A wound dressing can function in different ways, including removing wound exudates, keeping the wound environment moist, preventing infections, protecting from external hazards, as well as promoting the reconstruction of the wound by influencing epidermal migration, angiogenesis, and tissue formation [102]. In 2019, antimicrobial wound dressings was a $570M market with a compound annual growth rate (CAGR) of 9.1% predicted from 2020 to 2027 [19]. There are numerous commercial wound dressings, with a 12.2% CAGR predicted for 2022–2029 [33]. The ability of a dressing to absorb, hold, and kill bacteria present in infected wound fluid can work in tandem with systemic antibiotics, which may not reach the wound surface [42].

## 5. Silver-Based Dressings

**Figure 2:**
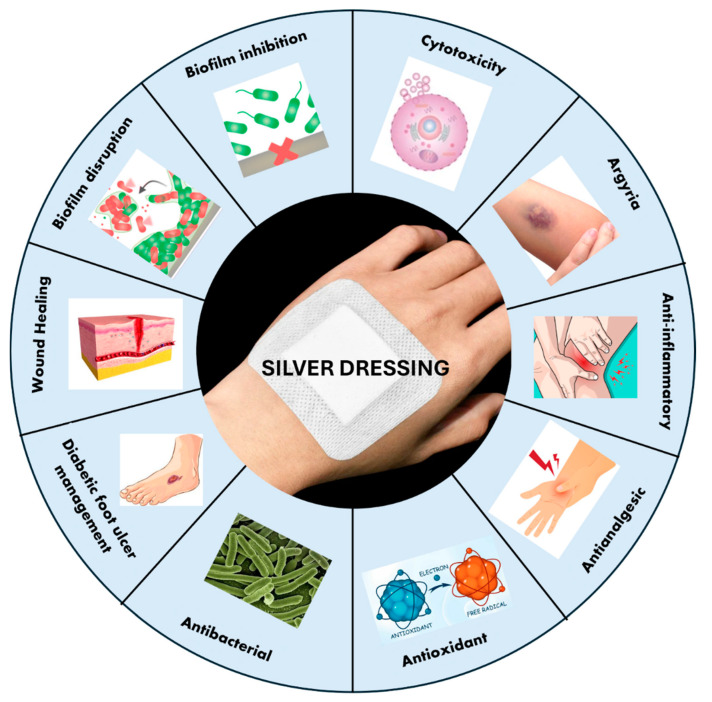
Possible roles played by a silver-based wound dressing, based on studies of silver.

Silver is often used as an antimicrobial in wound dressings, gels, lotions, and coatings for medical devices. Based on the FDA 510K Premarket Information, there are about 123 silver wound dressings. Figure 2 shows the various possible aspects of a silver wound dressing that are relevant in designing these dressings. These particular effects of silver are usually obtained from model studies and not necessarily shown with specific silver wound dressings [103]. Though silver is effective against both Gram-positive and Gram-negative bacteria, activity towards Gram-negative bacteria is more pronounced, primarily because of the thinner peptidoglycan layers in the Gram-negative bacteria [9,33,104]. The silver mechanism of action is mediated through silver ions, which bind to the tissues and intracellular proteins (N, O, or S functionalities), bacterial DNA, and RNA influencing respiratory chains. Cellular toxicity can be mediated through reactive oxygen species (ROS), and structural changes become possible in cell walls and intracellular and nuclear membranes. As an effective antimicrobial, silver should be helpful for the reduction in secondary infections [24]. Silver is shown to have anti-inflammatory [26] as well as anti-angiogenic [105] effects, affects the immune response [28], and can act as an antioxidant [106]. Early intervention with a silver dressing may decrease biofilm formation, although it is unclear what silver dressing alone can do if the biofilms are already formed [41,53].

Table 1 is a summary of the silver dressings described in this review (information primarily obtained from the web) and the in vitro and in vivo. studies of these dressings The in vitro models include the colony biofilm model and Duckworth Biofilm Device. Since pig skin is representative of human skin with similar anatomies, ex vivo porcine skin has been used in the in vitro model systems. Limitations of the in vitro biofilm studies are that they lack the dynamic and complex nature of the wound system, including the host immune system.

Animal models include the mouse chronic wound model, rabbit ear wound healing model, and porcine models [91]. Even though no animal model captures all the features of human skin, the wound reconstruction process, and the immune response, the porcine models come closest to that of humans [107]. The similarities between humans and pigs are the dermal to epidermal thickness (though the dermis in pigs lacks eccrine glands), lack of panniculus carnosus (wound closure is achieved by reepithelization), sparse body hair with hair follicles, and immune systems (though with a few disparities). In addition, other similar morphological characteristics of porcine skin with human skin include minimal hair coat, epidermal turnover time, a well-differentiated papillary body, and elastic tissue, as well as similar mechanisms of erythema and wound exudates [108]. However, the comorbidities in humans such as diabetes, atherosclerosis, lifestyles, and the healing of human wounds over long time frames such as months to years cannot be modeled readily in animals [1,17]. Animal models that take into account comorbidities include ischemic wounds, ischemic reperfusion wounds, pressure ulcers, and diabetic wounds [1,109,110].

In order to study how wound dressings affect biofilms, scanning electron microscopy (SEM) is useful [25]. The EPS layer can be studied by visualization and staining [1]. Other methods to study biofilms include light microscopy, confocal microscopy, and fluorescence microscopy, using selective staining agents [91]. Colony-forming unit assays are also commonly examined to investigate biofilms in wounds, but it should be noted that persister bacteria may be non-culturable [80].

Important characteristics of silver-based dressings are as follows: (1) how quickly the silver is released, (2) how long the silver release lasts, (3) the concentration of the silver being released, (4) the efficiency of the silver reaching the bacteria, (5) if other actives present in the dressing are being released into the wound, and (6) the role played by the matrix of the dressing. Silver is released from the dressing on contact with exudate and wound fluid. Multispecies biofilms are more difficult to treat because of the virulence of the organisms due to interspecies competition, leading to proteases and cytotoxic molecules that degrade the wound [35,111]. An advantage of using silver is that biofilm bacteria that survive silver are “damaged” and more susceptible to antibiotic attacks [25]. In treating biofilm-infected wounds, silver has difficulty penetrating the EPS layer [105,112].

Investigations of the *Pseudomonas putida* biofilms at three different levels of maturity show that mature biofilms have considerably reduced susceptibility to silver as compared to immature biofilms [52,113]. Thus, it is possible that silver dressings may not be effective for wounds that have established biofilms [113].

### 5.1. Forms of Silver and Additives in Dressings

Typical forms of silver used in wound dressings include ionic silver, in its common +1 form, as well as higher valent silver, and metallic silver in bulk or nanoparticle morphology, with the latter chosen because the release characteristics can be enhanced as compared to metallic silver [25]. AgNP (silver nanoparticles) were found to be better prophylaxis of infection as compared to silver ion dressings [26]. Strategies for the delivery of AgNP via microneedles have been attempted, with the elimination of the bacterial burden after administration for 60 h in a rat skin model [19]. Nanoparticles have the potential to reach biofilms in deep tissues [16]. Studies have shown that some bacterial species, e.g., *Pseudomonas aeruginosa*, will release surfactant-like rhamnolipids that promote the dispersal of the biofilm so that bacteria can find new anchoring sites [16,17,114,115]. Given this knowledge, surfactant-based wound dressings along with silver have been developed [16]. A silver dressing with benzethonium chloride that can better disrupt biofilms as compared to silver-only dressing has been commercialized [31]. In addition, along with surfactants, chelating agents such as citrate and EDTA that can complex metal ions (e.g., Ca^2+^) and weaken the EPS layer are reported [29,31,33,116].

Silver sulfadiazine (SSD) dressings were the first commercial silver dressing; 1% SSD was first used in 1968 for infection minimization in burn wounds [117]. Silver sulfadiazine combines silver and antimicrobial sulfadiazine and has been shown to reduce the microbial burden in a rat burn model [26]. A surfactant-based wound dressing along with silver sulfadiazine has been shown to eradicate mature biofilms [118]. SSD needs to be changed twice daily, and there are also reports of more pain for patients [64]. This has led to the introduction of silver dressings with a more controlled release than SSD dressings, and these dressings do not need to be changed as often [77]. Silver, along with antibiotics (e.g., tetracycline, gentamicin), shows enhanced antimicrobial properties, and there has been a report of AgNP combined with aztreonam to disrupt *P. aeruginosa* biofilms [119,120,121].

### 5.2. Release Characteristics

The release characteristics of the silver into the wound environment are critical since it is necessary to kill bacteria but ideally with minimal collateral damage to the cells necessary for wound healing. The rapid release of silver from the SSD dressing in burns slows down epithelization and promotes scar formation, whereas the dressing with AgNP did not, indicating that the release characteristics of silver play a role in wound healing [122].

It is proposed that the ideal dressing should release 10–40 ppm (<60 ppm required for more resistant bacteria) in a sustained manner over days. In the lower part of this concentration range, silver may promote reepithelization since it will have lower cytotoxicity and prevent microbe contamination [33]. The idea is to have enough silver to kill bacteria but not cause cytotoxicity [112,122]. However, blanket recommendations for concentration ranges must be considered carefully since the environment into which the silver is released is critical. Since the wound environment will have proteins, the formation of silver–protein complexes will alter the release of silver from the dressing [30]. A related observation is that silver penetration into porcine skin was dependent not on the amount of silver in the dressing but on how much silver is released into a protein-rich medium [30].

How the protein-rich silver wound exudate deposits will release silver is not well understood [25,26]. However, there is the recognition that because of the wound exudate binding of the silver, the silver may need to be orders of magnitude greater in concentration for the manifestation of antimicrobial activity [25]. On the positive side, the silver bound by wound exudate and wound scale may release silver slowly and offer protection from cytotoxicity. If the silver wound exudate deposits do not release silver, then the dressings will not result in germ-free wounds. Wounds have complex three-dimensional topology, and the distribution of bacteria in polymicrobial wounds is not uniform. If silver is tied up with the exudate, the silver may not reach the bacteria in the deeper tissues of chronic wounds. All of these conflicting parameters explain why the amount of silver in the dressing may not correlate with wound healing activity [23].

Since the Ag release characteristics of the dressing and thereby performance depends on multivariate factors, including the silver content, composition of the dressing, nature of the substrate, as well as the surrounding medium in the wound [30], it is not surprising that in a rat partial-thickness burn study, different silver-based dressings showed better results during different phases of the healing process and influenced the closure of the wound, inflammation, collagen production, and scar formation differently [24].

### 5.3. Toxicity

The optimal performance of silver-based wound dressing on infected wounds will depend on how effectively the bacteria is killed and how that environment is sustained without interfering with the healing process [123]. Because of the cytotoxicity of silver, the use of silver-based dressings on non-infected wounds can have a detrimental effect [122]. There are reports of impaired in vivo wound healing with silver dressings [124,125,126,127]. Renal and hepatoxicity have also been associated with silver dressings. There are reports of silver causing oxidative stress and being correlated with oxidative stress in cell lines [128]. In vitro studies of dermal fibroblasts suggest that subtoxic concentrations of silver released from the dressings may induce senescence which can delay wound healing due to the pro-inflammatory phenotype of senescent cells [30]. Though systemic silver absorption is low, silver dressings applied to large surface area wounds or with infants may lead to argyria [26]. It can take several weeks for silver to disappear from the skin [112]. Silver resistance is rarely encountered due to its multimodal mode of antimicrobial activity [26]. The additives used in silver dressings such as surfactants can accumulate at the wound site and delay wound healing [16]. Surfactants demonstrate severe cytotoxicity (90%) and adverse effects on cell proliferation [34].

### 5.4. Role of the Dressing Matrix

The ability of wound dressing needs to be balanced with exudate management, without compromising antimicrobial properties. Wound dressing material can influence exudate management, debridement of wound debris during dressing change, and wound management [25,91,112]. There are a variety of substrates that are used in silver dressings. As a class, hydrophilic dressings will lose activity since they can get contaminated by the wound exudates, tand the silver becomes bound. Hydrophobic dressings will release silver slowly but may not get deactivated [25]. Gel supports release silver very quickly and can be useful for highly infected wounds, whereas silver that is matrix-bound releases silver more slowly. Gel-based wound dressings may need more frequent application. The wound exudates can cause the formation of necrosis/crusts that impair the healing process due to the prevention of cell migration and reepithelization, interfere with granulation, and prolong inflammation [24]. Dressings with carboxymethyl cellulose and hydrofiber can absorb wound exudate. Alginate dressings can promote better wound hydration and autolytic debridement [24]. Alginates can provide a moist environment, converting wound exudates into a gel [67]. Collagen-based extracellular matrix (ECM) substrates promote wound healing by stimulating proteins related to collagen type I, II, and V, and dermal fibroblasts [34,122], and reduce pain levels [34]. They provide a lowering of pH, promote bacteriostatic, and support tissue repair and replacement by the breakdown of ECM proteins and cellular content [34,129]. There is a possibility of hypersensitivity with these xenogeneic ECM dressing matrices [101]. Amongst the matrices for silver wound dressings are charcoal-containing dressings that reduce odor. Silicone and membrane matrices are gentle on the skin and can conform to different wound shapes and sizes [130].

## 6. Clinical Studies

Table 2 and Table 3 list the clinical studies with silver dressings, and several aspects need to be noted. First, it is difficult to compare different clinical reports. Second, for any particular study, the important issues to consider include the following:

- Treatment duration;
- Sample size and diverse demographics;
- Potential biases in the study, including where the funding is coming from;
- Safety profile of the dressing;
- Bacterial load, depth of wound;
- Consideration of both the patient and physician perspective;
- Statistical methods used to analyze results, i.e., are the results of statistical significance;
- Description of the limitations of the study;
- Comparison of what worked and what did not work provides insight;
- Placebo/control effects are not always studied, as in comparing two silver dressings;
- Time to heal for participants who did not heal during the study are often excluded.

These points are elaborated in Table 2 and Table 3. This discussion highlights some of the broader observations from Table 2. In clinical trials, the important issues are as follows: (1) Nature of trial (method of randomization: was allocation concealed, blinding to participants, care provider, assessor [131,132], setting, location, source of funding); (2) Participants, including number, sex, wound type, how the infection was determined, how long the infection lasted, wound size, wound duration, follow-up until wound healing, and comorbidities; (3) Intervention including the type of dressing, silver content/dosage, frequency of dressing changes, co-interventions uniformly to all groups; (4) Treatment of incomplete outcome data; (5) Drop-out rate should be < 20%; and (6) Similarity of patient groups at baseline.

The primary outcome for wound healing is the time to complete healing and is the only fact important for the patient. Wound healing trajectories (wound surface area/volume per unit time) provide important clinical information [46]. A 20–40% reduction in wound area between 2 and 4 weeks is a good predictor of healing [41]. Other important issues are the rates of wound infection as measured by localized pain/swelling, erythema, purulent exudate, and bacterial counts > 10^5^ CFU/mm^3^ of tissue. Multiple measurements during the healing process increase the chance of false positive results due to drawing inconclusive conclusions about efficacy. Several features are relevant for secondary outcomes. These include adverse events, the need for systemic antibiotics, pain, patient satisfaction (very important), health-related quality of life, length of hospital stays, and cost minimization.

Several suggestions for clinical use of silver dressings can be gleaned from Table 2. Use of silver dressing for wounds that are locally infected or contaminated with antibiotic-resistant pathogens or at risk of infection is recommended. The procedure suggested is that the wound be cleaned/debrided and treated with silver-based dressings for 14 days, and then assessed to figure out if the therapeutic goal is being achieved. If not, other strategies should be considered [52,130]. The hypothesis is that silver dressings may decrease the bacterial load to prevent the chronicity of the wound by reducing the inflammation, and then followed by other treatments to promote wound healing [50]. The silver dressing can get wounds unstuck in the inflammatory stage [48]. For infected wounds, early silver antimicrobial intervention and then the possible discontinuance of dressing is a strategy [44]. Application of silver dressings without debridement may lead to non-adherence of the dressing to the wound surface [101]. The age of the patient is relevant; long-term silver dressing use in elderly patients can lead to silver accumulation [51].

Within a clinical trial, there are often observations that the dressing is not working for a particular set of wounds. A possibility that has been pointed out is that the active element silver is not penetrating deeper into these wounds, where bacterial colonization has occurred [39]. This could occur because silver can readily precipitate in the wound fluid, and thus strategies to promote silver penetration deeper into wounds would be useful. The duration of the clinical trial varies in studies, with the optimal period being unclear [40]. Bacterial load in the presence of the same wound dressing is patient-dependent [47], making interpretations difficult as to the efficacy of the dressing.

There are several retrospective studies, which can be useful, but a cautionary note is that it can suffer from bias, and the control of confounding variables from the patient end is lacking [52].

Analysis of random controlled trials suggest that silver-based dressings or creams may not be clinically effective for the following: (1) contaminated/infected wounds; (2) preventing infection, and (3) promoting wound healing [131,132]. The VULCAN trial found no advantage of silver dressings for venous ulcers [45]. Silver dressings are not recommended by the International Working Group of Diabetic Foot Ulcers for routine ulcer management [133]. There was no evidence for healing in diabetic foot ulcers at the 12-week mark in the largest randomized controlled trial reported [56]. However, an international group of clinicians suggests that silver dressings have an important role in reducing bioburden in wounds and have implications for shorter hospital stays [134].

Table 3 deals with burn wounds. Typically, partial-thickness burns heal within 2–3 weeks, without significant scarring. An ideal burn wound dressing should prevent transdermal fluid loss, prevent infection, promote reepithelization, be cost-effective, lower pain and be comfortable to use, and not interfere with other treatment modalities [68,76]. Partial-thickness burns often present a dilemma of treatment with surgical intervention since some of these wounds may heal on their own. In these latter cases, moisture-retentive or occlusive dressings provide an alternate treatment route. Wound dressings that provide moist healing can prevent scab formation. The mortality rate in burn populations is 38–45%, and after antimicrobial therapy was introduced, this dropped to 14–25% [71]. Large amounts of exudates can increase bacterial load. Including silver in dressings as a prophylactic antimicrobial agent is of value [58,61]. It is difficult to compare different dressings for burn wounds because it is not easy to select burns with comparable depths for comparing different dressings; laser Doppler imaging is a technique to measure depth but is difficult to use clinically [72].

## 7. Concluding Thoughts and Future Scope

Antimicrobial action can be a helpful intermediary step in the process of wound healing, though the critical issue is the impact of the dressing on the complete wound healing process. Dressings that release silver rapidly are preferable for wounds with heavy exudate and bacteria. Silver released over several days is relevant for moderate to severe pathogenic bacteria. Low silver content dressings can be helpful for low-grade infections or as a barrier to infections. Highly infected wounds can benefit from silver dressings since killing bacteria is more important than cytotoxic damage. Silver dressings with additives such as surfactant and chelating agents can be useful for biofilm-infected wounds. Silver dressings are relevant for infected non-healing wounds and not for well-managed and already healing wounds, where silver toxicity can be detrimental to the rapidly proliferating fibroblasts and keratinocyte cells in the granulation and reepithelization stage. Contact between the dressing and the wound is important, thus attention should be paid to the conformability of the dressing. Also, how the silver and the additives are spread on the dressing is important. There may not be a single ideal dressing for the entire wound healing period. New technologies for silver delivery are required for silver in the wound dressings to penetrate, unchanged, deeper into the wound to address the varying distribution of pathogens in the wound. Increasing the analgesic and anti-inflammatory properties of silver dressings would be useful. No one treatment can likely address all the deficits in a hard-to-heal wound. In clinical studies, the end points need better coordination between different investigations, with wound healing being the ultimate focus. There needs to be more randomized control studies to assess the advantages/disadvantages of different dressings.

## Figures and Tables

**Table 1:** Descriptions of silver-based commercial dressings and their properties in in vitro studies (in alphabetical order).

Dressings	Silver Content	Description	Notable Characteristics	Findings Related to Biofilms
Aquacel^®^ Ag^+^ Extra^™^ (ConvaTec)	0.17 mg/cm^2^	Hydrofiber™ Technology and Ag^+^ Technology—Two layers of a needle-punched nonwoven fleece of sodium silver CMC (carboxy methyl cellulose) fibers enhanced with EDTA and benzethonium chloride stitched with a high-purity cellulose thread. CMC forms a gel in contact with wound fluid.	Dressing formulated for disruption of mature biofilms [29]. Combinations of metal chelators (binds ions) and surfactants (softens EPS layer) [29]. Negative effect on fibroblast proliferation [23]. EDTA+BT may cause cytotoxicity [28].	Biofilms were made with a colony-drip flow reactor. *Pseudomonas aeruginosa* (PA) biofilms (72 h, confirmed by SEM) were exposed to the dressing for 24 h and had 8.8 log_10_ bacteria as compared to the control with 9.2 log_10_ bacteria (not significant). With the 24 h biofilm, 9.2 log_10_ bacteria for the control versus 6.6 log_10_ bacteria with the dressing were observed [23]. Biofilms were grown in a CDC reactor for 72 h. Dressings were effective against *Staphylococcus aureus* (SA) and PA biofilms and *Candida* (CA) yeast biofilms (high levels of extracellular material). Multispecies bacteria (SA, PA, CA) were grown on porous polycarbonate in a CDFR flow reactor for 72 h, and the dressing was found to be effective [30]. With SA and PA biofilms a 4-log_10_ decrease over 5 days (9 log_10_ to 4 log_10_, 1 log_10_ every day), the addition of bacteria on 5th day did not result in biofilm re-formation [31]. In a porcine ex-vivo model, a 72 h grown biofilm was applied to the skin, and cultured for another 24 h. Biofilm viability was 13% as compared to 77% for the control [28]. In an in vivo murine model, colony biofilm was grown (72 h) on membranes and applied to the full-thickness excisional wound and, after 3 days of observation, no reduction in wound area or epithelization as compared to controls was noted [28]. PA- and PA+SA-infected dermal punch wounds were made in rabbit ears. Test dressing decreased bacterial counts and improved wound healing (*p* < 0.05), but dressing was not effective against the SA within the wound [32].
Aquacel Ag Extra (ConvaTec)	1.2% *w*/*w* ionic silver	Composed of sodium carboxymethylcellulose (CMC) fibers impregnated with ionic silver, enforced within strengthening fibers.	Ag^+^ released into broth (TSB) 28.1 ± 1.4 μg/mL in 24 h and 1.4 ± 0.1 μg/mL over 7 days [25]. In cell culture media with 10% FBS, 18.1 μg/mL Ag^+^ released after 72 h of agitation [30]. 107 ppm Ag released into de-epithelized porcine skin explants in 24 h [30]. Acute cytotoxic response towards HaCaT keratinocytes and primary human dermal fibroblasts [30].	Extracellular polymeric substrate (EPS) embedded colonies of 45–600 μm for PA (7.6 log_10_ bacteria) and for MRSA (6.2 log_10_ bacteria), colony thickness of 54–88 μm were formed. An *E. coli* biofilm (5.6 log_10_ bacteria), 10–70 μm in diameter with 5–12 μm thickness, was formed. Upon exposure to the wound dressing, there were 2.8 log_10_ and 1.5 log_10_ decrease for PA, 2.9 and 1.6 log_10_ decrease for MRSA, and 3.5 and 1.8 log_10_ decrease for E.coli biofilms over 24 h and 7 days, respectively [25]. Biofilms were studied with an in vitro Drip Flow reactor. Dressing impeded new biofilm formation for PA (4.3 log_10_ decrease) and SA (2.3 log_10_ decrease). For SA and PA mixed species biofilms grown on hydroxyapatite for 3 days prior to treatment, exposure to dressings for 24 h resulted in 3.4 log_10_ decrease for SA and 1.3 log_10_ decrease for PA [33]. Deep reticular dermal wound infected with MRSA for 72 h to form biofilm in a porcine model, debrided, and treated with wound dressing. A 1 log_10_ decrease in MRSA on day 4, 2 log_10_ decrease in MRSA on day 8, and day 11 as compared to control were observed. Day 11 observations were 90% reepithelization, with marked angiogenesis and white cell infiltration, and granulation tissue formation approaching 76–100% [34].
Acticoat^™^ 7 (Smith & Nephew)	1.70 mg/cm^2^	Two rayon/polyester non-woven inner cores laminated between three layers of nanocrystalline silver-coated high-density polyethylene mesh, designed to be the barrier against bacterial invasion.	Ag^+^ release in broth (TSB) 11.7 ± 0.8 µg/mL in 24 h and 8.0 ± 0.6 µg/mL in 7 days [25]. In cell culture media with 10% FBS, 18.1 μg/mL Ag^+^ released after 72 h [30]. Ag^+^ in de-epithelized porcine skin explants was 143 ppm Ag in 24 h. Acute toxic response towards HaCaT keratinocytes and primary human dermal fibroblasts Fibroblast proliferation decreased [23].	In a Drip Flow reactor, dressing impeded new biofilm formation for both PA and SA. With mature SA and PA mixed species biofilms grown for 3 days, exposure to dressings for 24 h led to a 3.4 log_10_ decrease for SA and a 1.3 log_10_ decrease for PA [33]. PA biofilm had 7.6 log_10_ bacteria, MRSA biofilm had 6.2 log_10_ bacteria, and *E. coli* biofilm had log 5.6_10_. For the PA biofilm, there was a 4.2 log_10_ decrease in bacteria in 2 h, and a 4.5 log_10_ decrease after 7 days; for MRSA, there was a 4.6 log_10_ decrease for both 24 h and 7 days. For the *E.coli* biofilms, there was a 4.8 and 5.0 log_10_ decrease in bacteria over 24 h and 7 days, respectively [25]. There was significant silver accumulation in the biofilms [25]. Dressing did not destroy biofilms for MRSA and PA [31].
BDWG	No silver		Polyethylene glycol (PEG) gel containing benzalkonium chloride (0.13 wt%), citric acid (3.41%), and sodium citrate (3.57%). Manifested severe cytotoxicity towards fibroblasts, and fibroblast proliferation was compromised [34].	Using an in vitro Drip Flow reactor, the dressing impeded new biofilm formation for both PA and SA. With SA and PA mixed species biofilms exposure to dressings for 24 h led to a significant decrease in bacteria (5.9 log_10_ decrease for SA and 6.6 log_10_ decrease for PA) [33]. A deep reticular porcine dermal wound model infected with MRSA (72 h biofilms) was debrided and then treated with wound dressing. A 2 log_10_ reduction in MRSA counts was observed after 4 days, and a 3 log_10_ decrease after 8 days and 11 days. The wound approached 80% reepithelization on day 11, along with marked angiogenesis, and granulation tissue formation approached 76–100% [34].
Biatain Ag (Coloplast)	1 mg/cm^2^	Hydrophilic polyurethane hydro cellular, silver ions in the form of a complex (formerly Contreet Foam).		
Biatain Alginate Ag (Coloplast)	0.95 mg/cm^2^	An alginate dressing consists of calcium alginate, carboxymethylcellulose (CMC), and an ionic silver complex.		
Contreet Foam (Coloplast)	1 mg/cm^2^	A soft hydrophilic polyurethane foam containing silver. Foam bonded to a semi-permeable polyurethane film. Silver ions are hydroactivated in the presence of fluid or wound exudate. In vitro studies show that silver release is sustained for 7 days, and the release is proportional to the amount of exudate absorbed.		
Exufiber Ag^+^ (Mölnlycke)		A dressing made with PVA and hydroxypropyl cellulose gel with Ag_2_SO_4_		Biofilms were grown on plates in a CDC reactor for 72 h and exposed to dressings for 24 h. Dressings were effective against SA and PA biofilms separately; for multispecies biofilms, the dressings were not effective [35].
Ialugen SSD	120 µg/cm^2^	A dressing impregnated with cream containing Na hyaluronate, SSD, macrogol 4000, and 85% glycerol.	Silver in de-epithelized porcine skin explants: 188 ppm Ag in 24 h [30]. Dressing showed acute toxic response towards keratinocytes and primary human dermal fibroblasts.	
Kerracel^®^ Ag (3M)	0.2 mg/cm^2^/1.7% (*w*/*w*) Ag Oxysalts (Ag_7_NO_11_) [28]	A dressing formulated with Ag oxysalts™, a non-woven sterile wound dressing using a mix of 100% carboxymethylcellulose (CMC), cellulose fibers, and silver to create a barrier against bacterial growth for as long as 7 days.		Biofilms were grown in a CDC reactor for 72 h. Dressings were effective against SA and PA biofilms separately and ineffective against *Candida* yeast biofilms (24 h exposure). With multispecies biofilms on nonporous polycarbonate, the dressing was very effective but not so when the biofilms were grown on porous polycarbonate (better representation of hard-to-heal exudating wound) [35]. A 72 h grown biofilm was placed in porcine ex vivo skin, cultured for 24 h to allow for attachment, and dressing was applied for 24 h. Dressing led to 14% biofilm viability as compared to 75% for control. A 72 h colony biofilm grown on membranes was applied to the full-thickness excisional wound in a murine model. Exposure to dressing for 3 days led to a smaller wound area in PA and SA biofilms, although not statistically significant. Wound area and reepithelization were 34% for PA, control 15%, 31% for SA, and 14% for control. Macrophage reduction within the granulation tissue in SA biofilm-infected wounds was significant [28]. The study used an in vitro Drip Flow reactor. Dressing impeded new biofilm formation for both PA and SA. However, for SA and PA mixed species, exposure to dressings for 24 h led to a 0.8 log_10_ decrease for SA and a 0.3 log_10_ decrease for PA [33].
Maxorb Ag^+^ Extra (Medline Industries)		A dressing that uses CMC and calcium alginate with AgNaZrPO_4_		Biofilms were grown in a CDC reactor for 72 h. Exposure to dressing for 24 h indicated that the dressings were effective against SA and PA biofilms separately and ineffective against *Candida* yeast biofilms. For a multispecies biofilm grown in a CDFR flow reactor biofilm on porous polycarbonate (better representation of hard-to-heal wound), the dressing was not effective [35].
Mepilex Ag (Mölnlycke)	1.25 mg/cm^2^	A dressing composed of absorbent polyurethane foam with a composite of silver and activated carbon. The silver source is silver sulfate which releases silver ions. The outer film is permeable to water vapor and impervious to liquids [24]. There is always a layer with silicone adhesives that stays in contact with the wound.		The study was with partial-thickness burn in rats. The effect of dressing on the inflammatory phase (7 days), proliferative phase (14 days), and remodeling of the wound (30 days) was examined. Necrosis was noted, possibly due to poorer wound hydration due to the absorption of the wound exudate. Observations were as follows: higher inflammatory infiltration of healing PMN cells during the 7 days; on day 14, less hemorrhage, more angiogenesis, and more granulation tissue; and on day 30, more fibroblasts to promote wound closure [24].
Primatrix Ag (Integra)	165 µg/cm^2^	A dressing containing fetal bovine Type III collagen and silver.		A deep reticular porcine dermal wound was infected with MRSA for 72 h to form a biofilm, debrided, and then treated with wound dressing. There was a 2-log_10_ decrease reduction in MRSA counts obtained from biofilms on days 4, 8, and 11 as compared to the controls. The wound approached 85% reepithelization on day 11. Marked angiogenesis, along with white cell infiltration, was observed on day 11. Granulation tissue formation approached 76–100% on day 11 [34].
Procellera™ (Vomaris)	Ag: 0.9 mg/cm^2^ Zn: 0.3 mg/cm^2^	Microcurrent-generating antimicrobial wound dressing consists of a matrix of alternating silver and zinc dots held in position on a polyester substrate with a biocompatible binder.		Antibacterial efficacy against β-lactamase bacteria, multidrug-resistant bacteria, and MRSA. Ineffective with Enterococcus bacteria [36].
Promogran™ PRISMA (3M)	1% silver-ORC contains 25% *w*/*w* ionically bound Ag. Ag: 20 µg/cm^2^	A sterile, freeze-dried composite of 44% oxidized regenerated cellulose (ORC), 55% collagen, and 1% silver-ORC.	Did not inhibit dermal fibroblast growth [23].	PA biofilms made with colony-drip flow reactor (72 h, confirmed by SEM) upon exposure to dressing for 24 h led to 7.8 log_10_ bacteria as compared to the control of 9.2 log_10_, not a significant effect. Results for less mature 24 h biofilm were 9.2 log_10_ bacteria with control gauze versus 6.5 log_10_ with dressing. Gentamycin-treated biofilm reduction in BPA (bacterial proteases) was 77% compared to the control, possibly due to the ORC/collagen matrix [23]. Not very effective against MRSA [34].
Polysheet metallic Ag	<25 µg/cm^2^	A dressing with polyelectrolyte and polyvinyl alcohol polymeric sheet containing ionic and metallic silver.		Studies were conducted with an in vitro Drip Flow reactor. Dressing impeded new biofilm formation for PA but not for SA. For SA and PA mixed species biofilms, exposure to dressings for 24 h led to a 0.5 log_10_ decrease for SA and a 4.6 log_10_ decrease for PA [33].
PU Foam Ag salt	0.35–0.4 mg/cm^2^	A dressing with metallic silver and starch copolymers on a polyurethane membrane.	The amount of silver released into the broth (TSB): 14.7 ± 0.7 µg/mL Ag release in 24 h and then drops to 2.6 ± 0.1 µg/mL in 7 days (TSB).	Biofilms were grown in a Drip Flow reactor. The dressing was effective for thwarting new biofilm formation for PA but not for SA [33].
Silvercel (3M)	740 µg/cm^2^ to 863 µg/cm^2^, diffuse coating of Ag, (Ag^+^ or Ag coated fibers [30] 9 wt% silver in dressing [24]	A dressing composed of nonwoven hydroalginate, calcium alginate, guluronic acid (high-G) strength of 32%, sodium carboxymethylcellulose (8%), and nylon fibers (51%) covered with elemental silver (9%).	Silver in the de-epithelized porcine skin explants—111 ppm Ag in 24 h Acute toxic response towards keratinocytes and primary human dermal fibroblasts.	With dressing, the following were observed: a 1.9 log_10_ decrease in 24 h; after 7 days, a 0.9 log decrease for PA; and 2.6 and 1.6 log_10_ decrease for MRSA biofilms, and a 3.1 and 1.4 log_10_ decrease for E.coli biofilms [25]. Did not destroy biofilms for MRSA and PA [31]. In a study involving partial-thickness burns in rats, the dressing exhibited a decrease in necrosis, wound exudate, odor, as well as more granulation tissue helping wound healing. The presence of alginate possibly promoted better wound hydration and autolytic debridement [24].
Silverlon (Silverlon)	5.46 mg/cm^2^	A dressing with silver on nylon cores.	The release of Ag into broth (TSB) was 8.1 ± 0.4 µg/mL in 24 h and 13.9 ± 0.7 µg/mL in 7 days.	With dressing, a 1.2 log_10_ decrease in PA biofilms was observed over 24 h, and after 7 days, a 2.9 log_10_ decrease; there were also a 1.6 and 2.3 log_10_ decrease in MRSA biofilms, and a 1.0 and 3.0 log_10_ decrease for E.coli biofilms for 24 h and 7 days [25].
Silver sulfadiazine	1 wt% micronized silver sulfadiazine in gel	A gel with stearyl alcohol, polyethylene glycol hexadecyl ether, liquid petrolatum, propylene glycol, methylparaben, propylparaben, butylhydroxytoluene, and purified water.		Partial-thickness burns were induced in rats, and the wounds were monitored during the inflammatory phase (7 days), proliferative phase (14 days), and remodeling phase (30 days). Dressing increased necrosis, possibly because the gel did not promote the hydration of the wound bed [24].
Tegaderm Ag mesh (3M)	8 mL of silver per gram of dressing	Particles of silver sulfate are coated on the surface of cotton fibers. When wound exudate, sterile normal saline, sterile water, or liquid hydrogel comes in contact with the dressing, the silver sulfate dissolves, releasing silver ions in the dressing rapidly and over time.		
UrgoClean Ag (Urgo Medical)		A dressing with lipid-colloid and poly-absorbent fiber with Ag_2_SO_4_.		Biofilms were grown in a CDC reactor for 72 h. The dressing was effective against SA and PA biofilms separately but ineffective against the *Candida* yeast biofilms. For the multispecies biofilms (SA, PA, CA) grown in a CDFR flow reactor on porous polycarbonate (better representation of hard-to-heal wound), the dressing was not effective [35].
Urgotul Ag (Urgo Medical)	3.5% ionic Ag [37]	Non-occlusive, non-adhesive, flexible lipid-colloid dressing comprising a polyester mesh impregnated with hydrocolloid and petroleum jelly particles and silver.		

**Table 2:** Clinical Studies: Chronic Wounds (in chronological order).

Dressings	Clinical Method Summary	Quantitative Results	Year [Ref.]
Contreet Foam	Uncontrolled open study. Treatment of bacteria-infected chronic venous leg ulcers in 25 patients over four weeks. Assessment: healing in terms of wound-bed tissue composition, odor, pain, dressing performance, and effect on the per-ulcer area.	4 weeks: A mean reduction of 56% in the ulcer area (15.6 to 6.9 cm^2^) was noted. Week 1 observations were a mean reduction of 25% in granulation tissue from dull to healthy and that wound odor was significantly reduced. Half of the patients showed an increase in ulcer area after the removal of the Ag dressing (after 4 weeks).	2003 [38]
Contreet silver-based foam dressing as compared to a control dressing (Allevyn Hydrocelluar)	A multicenter study (15 centers in 7 countries). Open block-randomized and controlled 4-week study of 129 patients (Contreet: 65, Allevyn: 64) with colonized chronic venous leg ulcers.	A decrease in odor was noted after 1 week of treatment for 83% with the Contreet versus 53% in the control group. Lower maceration was observed after 4 weeks in the Contreet foam (37%) as compared to control (48%). A 45% median reduction in ulcer area was observed as compared to 25% in the control group, suggesting a faster healing process.	2005 [39]
Silvercel (silver-hydroalginate) compared with control Algosteril (calcium-hydroalginate)	A multicenter (13 centers) randomized, two-arm parallel-group study over 4 weeks of 99 patients (51 test group, 48 control group), with venous leg ulcer (71) or pressure ulcer (28); assessment performed over 4 weeks.	Fewer patients developed clinical infection (33%) compared to control (46%, *p* = 0.223). No patient in the test group required antibiotics as compared to the control (10.5%). Greater wound closure rate for the test group (0.32 ± 0.57 cm^2^/day) as compared to control (0.16 ± 0.40 cm^2^/day, *p* = 0.024). Reduction in wound severity score was greater in the test group (−32 ± 17%) as compared to the control group (−23 ± 25%; *p* = 0.034). The total modified ASEPSIS (wound scoring method) score over 14 days did not significantly differ between the test (104.2 ± 72.8) and control groups (95.4 ± 62.2; *p* = 0.791).	2005 [40]
Contreet Foam Outcome Program Comparison with Aquacel Ag, Actisorb, Acticoat Control-local best practice	Randomized controlled trial: A total of 619 patients with ulcers of varying etiologies were treated for four weeks; patients were either treated with the silver foam dressing (326 patients) or with local best practice (293). The objective was to assess the effects on wound area reduction, slough and maceration, exudate level, overall wound progress, exudate handling, ease of use, odor, pain, time spent on dressing changes, and mean wear time of the dressing.	Median ulcer area reduction upon final visit: Ag foam—47.1%, control—31.8% (*p* = 0.0019). Mean slough on the final visit: Ag foam—7%, control—8.9%. Mean macerated peri-ulcer skin: Ag foam—10.9%, control—16.7% (*p* = 0.0383). The odor was absent within 1 week. Superior exudate handling as compared to other Ag dressings was noted.	2006 [41]
Aquacel (1.2% ionic silver, AQ) and Algosteril (Calcium Alginate, CA)	A prospective, stratified, randomized, open-labeled, controlled, multicenter study, diabetic patients with non-ischemic Wagner Grade 1 or 2 diabetic foot ulcers (>1 cm^2^ area). A total of 134 patients’ wound dimensions were measured at 0, 4, 8 weeks, and upon healing. Standardized surgical debridement and callus removal were performed.	AQ-dressed ulcers showed a depth reduction of 0.25 ± 0.49 cm compared to 0.13 ± 0.37 cm in the CA-dressed ulcers (*p* = 0.04), An 8-week ulcer area reduction of 58.1% (AQ) vs. 60.5 (CA) (*p* = 0.948) was noted. The AQ group showed a healing speed of 0.29 ± 0.33 cm^2^ per week, compared to 0.26 ± 0.90 cm^2^/week for the control (*p* = 0.993). The 100% healing time was marginally lower for AQ (53 days) as compared to CA (58 days) (*p* = 0.34). Infected ulcers had a more favorable outcome with AQ vs. CA with systemic antibiotics.	2007 [42]
Urgotul Ag vs. Urgotul	Open-labeled, randomized controlled trial studying venous leg ulcers with heavy bacterial colonization in 102 patients, where 80% of the wounds were not progressing with the previous treatment.	Week 0: Mean ulcer area 20.0 ± 17.8 cm^2^. Week 4: Mean surface area decreased by 6.5 ± 13.4 cm^2^ (median: 4.2 cm^2^) and 1.3 ± 9.0 cm^2^ (median: 1.1 cm^2^) in Ag dressing versus control groups, respectively (*p* = 0.023). Week 4: Bacterial colonization was not clinically observed in 39.2% of Ag dressing versus 16.7% in the control group.	2008 [43]
Group 1: Acticoat; Group 2: Comfeet Ag hydrocolloid/Biatain Ag polyurethane foam; Group 3—Aquacel Ag	Prospective, comparative study on 75 patients, with 25 in each group. Wounds: leg ulcers, pressure ulcers, diabetic foot ulcers, and post-traumatic ulcers. All wounds showed clinical signs of infection.	Resolution of clinical signs of infection: Group 1—2.52 ± 1.29 weeks, Group 2—3.88 ± 0.44 weeks, Group 3—3.80 ± 0.58 weeks No clinical sign of infection: Week 2—60% for Group 1, 4% for Group 2 and 8% for Group 3 Fewer treatments were required in Group 1 to eliminate infection.	2008 [44]
Aquacel^®^ Ag, Acticoat™, Acticoat™ 7, Acticoat™ Absorbent, Contreet^®^ Foam, Urgotul SSD versus non-silver dressings	In a multicenter study, 213 patients with active ulceration of the lower leg were presented for >6 weeks (107 patients had a random assignment to Ag dressings). The focus was on assessing the effectiveness of silver-donating antimicrobial dressings as a category.	No significant difference in the proportion of ulcers healed at 12 weeks: 59.6% for silver and 56.7% for control dressings. The overall median time to healing was 67 days for antimicrobial dressings and 58 days for the control group (*p* = 0.048). No significant differences were observed between the groups in terms of health-related quality of life. A significantly higher cost was associated with silver dressings.	2009 [45]
Acticoat™ compared with Iodosorb cadexomer iodine	The study used a parallel-group, open-labeled randomized controlled trial (TBSA). Participants had a lower leg ulcer with an ankle brachial pressure index of 0.6 or above, the wound was 15 cm or less in diameter and had evidence of critical colonization. Sample of 281 participants, with 140 for Acticoat and 141 for Iodosorb, in a 12-week study.	Similar overall healing rate for silver dressing (64%) compared to iodine (63%), with a similar daily healing rate. Acticoat and Iodosorb were comparable in terms of the number of wounds healed. Acticoat was associated with a quicker healing rate during the first 2 weeks of treatment, but this was not sustained beyond that time. Silver dressing showed a significantly higher rate of healing for wounds that did not heal in the 12 weeks (larger, older wounds).	2010 [46]
Tegaderm Ag mesh dressing compared to silver sulfadiazine cream	Randomized clinical trial in a single hospital for 8 weeks with 40 patients for treating pressure ulcers; study conducted detailed microbiologic studies of the wounds.	SSD cream application is labor-intensive and expensive. The mean healing rate in the eighth week was lower (25.06%) in the SSD group as compared to the mesh group (36.95%, not statistically significant, *p* = 0.507). Pressure Ulcer Scale for Healing (PUSH) score, an indicator of ulcer severity, was higher initially as well as in the eighth week in the SSD group compared to the mesh group (*p* = 0.473). Difficult to conclude anything definitive from the microbiologic studies and needs statistical analysis.	2011 [47]
Urgotul Ag versus Urgotul (without Ag)	This was an open-labeled randomized controlled trial (not double-blind) for 4 weeks (followed for additional 4 weeks). Patients with venous leg ulcers (VLUs) showed at least three out of five clinical signs of bacterial colonization. A total of 99 patients (51 with silver and 48 control) participated in the study.	At week 4, the median wound closure rate was 0.145 cm^2^/day for Urgotul Silver vs. 0.044 cm^2^/day for the control group at week 4 (*p* = 0.009). At week 8, the median decrease in wound size was 5.9 cm^2^ for the Urgotul Silver group compared to 0.8 cm^2^ for the control group (*p* = 0.002). 55% of ulcers showed a >40% decrease in wound area for the Urgotul Silver group compared to 35%% for the control group. At week 4, 39.2% of ulcers showed no clinical signs of colonization as compared to 16.7% in the control group. Local adverse events were comparable in both groups.	2012 [48]
Aquacel Ag dressing compared to Urgotul Ag	Two-arm parallel multi-center open-labeled randomized controlled clinical trial for 8 weeks with 281 patients with chronic venous leg ulcers across 43 centers in multiple countries.	After 8 weeks, there was a relative wound size reduction of 49.65% ± 52.53% in the Aquacel group as compared to 42.81% ± 60.00% in the Urgotul^®^ group (*p* = 0.3158). At week 8, 39.5% of ulcers in the Aquacel group showed no clinical signs of heavy bacterial colonization, along with 32.5% of ulcers in the Urgotul^®^ Silver group (no significant difference). A total of 15% of subjects in the Aquacel group had healed ulcers, while 15.9% of subjects in the Urgotul^®^ Silver group had healed wounds (*p* = 0.0899). The inclusion of a placebo/control group would have been useful.	2012 [49]
Biatain Ag vs. Biatain	The study was a double-blinded controlled study with 181 patients (87 control) and conducted across 38 centers in five countries. Patients with venous or predominantly venous leg ulcers were recruited. The 6-week treatment period was followed by a 4-week open study with only Biatain; observations on days 0, 28, 42, and 70.	Biatain Ag demonstrated a greater wound area reduction (42%) compared to Biatain after 6 weeks of treatment (35%) (*p* = 0.0853). This would be more significant if older, larger ulcers were considered. Healing rate: Biatain Ag showed a Gilman rate of 0.67 mm/week compared to 0.53 mm/week for Biatain (French group: control 0.33 mm/week) (*p* = 0.0852). Both groups reported similar frequencies of local inflammatory signs after 6 and 10 weeks of treatment. Adverse events (maceration, eczema, pain, and burn) were observed in six events in Biatain Ag versus four in Biatain. A country-wise discrepancy was evident in the study.	2014 [50]
Aquacel Ag	The study was designed to evaluate the systemic absorption of silver in patients (criteria: silver levels > 0.5 µg/mL) with chronic inflammatory wounds and its association with silver toxicity. The study was a longitudinal, observational, multicenter, open-labeled pilot study using 40 elderly (patients mostly female, average age 74.3 years).	Dressing changed every 2 days between the initial day and day 28 of the treatment period. Mean wound surface area reduction was 22.8% (*p* = 0.041), along with a decrease in the fibrin percentage (beneficial for wound healing) between day 0 and day 28. Half the patients showed increased silver levels. There was no argyria or systemic toxicity. Elimination of silver from the body was slow and could result in cumulative toxicity, especially for elderly patients. The study recommends against long-term silver dressing use.	2018 [51]
Aquacel^®^ Ag^+^ Extra™ (All patients previously managed with traditional silver (26%), iodine (23%) or poly hexamethylene biguanide (PHMB) (11%) containing products or systemic antibiotics (12%))	The study recruited 65 patients with wounds ranging in duration from 1 week to 20 years (median duration: 12 months). 47 cases (72%) had stagnant wounds, and 15 cases (23%) had deteriorating wounds, while 3 wounds were not recorded; observations were made for 1–11 weeks. Participants also had clinical signs of infection or critical colonization.	Observations were as follows: 17% of wounds healed, 62% of wounds showed improvement, 14% of wounds remained the same, and 8% of wounds deteriorated. Moderate exudate (52% n = 24) and high exudate (37% n = 34) levels before treatment led to low (31%, n = 20) and moderate (43% n = 28) levels, respectively, after treatment. Biofilms were observed in 49% and slough in 42% of wounds. After applying the dressing, wound bed tissue was 63% granulated. Healthy wound bed tissues increased from 33% to 67% after treatment. Necrotic, slough biofilm reduced from 92% to 40% following treatment. Peri-wound skin health improved in 67% of cases.	2020 [27]
Acticoat™ Flex 7 (nano-Ag) with dressings without nano Ag	Retrospective study: 330 patients and 2242 patients control group in community centers with various types of wounds, including pressure injuries, diabetic foot ulcers, and venous leg ulcers (used Bates–Jensen Wound Assessment Tool).	Sustained silver release over 7 days. The mean time between dressing changes was 3.98 days vs. 1.87 days in control (*p* < 0.01), reducing nurse visits. The mean healing time for wounds treated with Acticoat 7 was significantly shorter (10.46 weeks) compared to wounds with control dressing (25.49 weeks). Only 0.9% of patients treated with Acticoat 7 dressing developed a systemic infection, compared to 3% in the comparative group. Potential for bias and no control for confounding variables, e.g., concurrent treatments.	2021 [52]
Biatain^®^ Ag Non-Adhesive Foam versus silver sulfadiazine	60 adult patients diagnosed with type 2 diabetes mellitus, with diabetic foot ulcers (DFU) area of at least 1 cm^2^ were recruited. Treatment Group: Biatain^®^ Ag Non-Adhesive Foam dressing applied at least every two days (38 patients). Control Group: 1% SSD cream applied once or twice per day (22 patients) A 4-week study, where debridement was performed during weekly visits, if necessary.	*Enterococcus faecalis* and *Staphylococcus aureus* were isolated from the wound culture in both groups. The proportion of the wound healed at week 4 in the SSD group was 27.00 ± 4.95%, while Biatain was 76.43 ± 7.41% (*p* < 0.0001). Silver foam facilitated wound closure faster than SSD in the patient population with HbA1c > 7% (59.94 ± 8.00% vs. 14.21 ± 3.72%, *p* = 0.027) and in patients with positive microbial isolates in their wound culture (60.87 ± 4.06% vs. 37.50 ± 5.89%, *p* = 0.020).	2021 [53]
Biatain alginate Ag versus gauze (some with iodoform)	40 patients in observation and 40 patients in the control group. Debridement and Biatain Alginate Ag were applied to the wounds. Dressing changed every 1 to 3 days. Assessment at 7, 14 days, and 1 month after treatment. The study observed the frequency of dressing changes, granulation tissue growth, wound formation, and healing time.	Pain score (VAS) was significantly different between Bitain and the control group (*p* < 0.05). Better outcomes in wound scar healing were observed as compared to the control group (*p* < 0.05). Enhanced granulation tissue growth was significantly higher in observation vs. control. Bacterial load was significantly lower than in the control group.	2022 [54]
Aquacel Ag^+^ versus Sorbact dressing (Cutimed Sorbact, Essity, retains exudate, no release of any antimicrobials)	Retrospective Patient Chart Audit with 350 patient charts: 200 with Aquacel Ag+ and 150 with Sorbact. Data analyzed separately for Germany and the US (DFU and venous leg ulcers).	Unclear why specific dressings were chosen for specific patients. Germany: Wound percent reduction and wound closure comparable; greater proportion of Sorbact users needed surgery (0 vs. 11%, *p* = 0.039). US: Wounds were worsening before the use of Aquacel (49% vs. 34%, *p* = 0.01), regression analysis suggests that it was 3.53 times more likely to have wound healed in Aquacel cohort (*p* = 0.033).	2023 [55]
Acticoat versus SoC	Prospective, open-labeled, randomized, placebo-controlled trial for acute diabetes-related foot ulcers, with 63 patients with Acticoat and 55 with SoC. The primary endpoint was the proportion of ulcers healed at 12 weeks.	Observation of ulcers healed at 12 weeks: 75% in the control group and 69% in the silver group (*p* = 0.49). No significant difference in complete ulcer healing (*p* = 0.53), osteomyelitis, need for amputation or antibiotic treatment between the silver and control groups.	2023 [56]

**Table 3:** Clinical Studies: Burn and Other Wounds (in chronological order).

Dressings	Clinical Method Summary	Quantitative Results	Year [Ref.]
Acticoat vs. 0.5% silver nitrate	Randomized 30 burn patients with symmetric wounds.	The frequency of burn wound sepsis (>10^5^ organisms per gram of tissue) was less in the Acticoat-treated wounds than in those treated with silver nitrate (5 vs. 16), as well as the observations of secondary bacteremia (1 vs. 5). Dressing removal was less painful with the Acticoat than with silver nitrate.	1998 [57]
Aquacel Ag	Phase II multicenter, open-labeled, noncomparative trial, where 24 patients with fresh superficial, mid-dermal, or mixed partial-thickness burns covering 5% to 20% of total body surface area (TBSA) were studied; trial lasted for 158 days.	Up to 77% of patients achieved over 95% re-epithelialization within 14 ± 3 days. The mean time for complete healing was 11.6 days.Significant reduction in pain between the baseline and post-burn days three and five. Positive reviews of conformability and ease of use were noted.	2004 [58]
Acticoat vs. SSD	Prospective Randomized Trial of adults with partial-thickness burns, with 14 patients, with a focus on pain management during dressing change.	Mean pain scores for wounds treated with Acticoat were significantly lower (3.2) as compared to those treated with SSD (7.9) (*p* < 0.0001).	2005 [59]
Aquacel Ag versus SSD	A comparative cost-effectiveness study comparing Aquacel Ag and SSD for superficial mid-dermal or mixed partial-thickness burns covering 5% to 40% TBSA (total body surface area). The 21-day study involved 84 patients, with 42 patients randomly assigned to each of the two treatment groups (mean age of 26.8 years, and 69.5% were men).	Aquacel^®^ Ag dressing had 73.8% of patients achieving full re-epithelialization, compared to 60.0% achieving full re-epithelialization in the silver sulfadiazine group (not significant, *p* = 0.222). Silver sulfadiazine was found to have significantly greater flexibility and ease of movement. Adverse events were comparable between the two dressings, though Aquacel was associated with lower pain Total cost with Aquacel was found to be less than SSD.	2006 [60]
Acticoat versus SSD	Multi-center randomized experimental design with blinding and positive parallel control. Work was performed at four burn centers across the country, with 98 patients with 166 residual wounds, comprising 79 men and 19 women, aged 18–63 years, with an average burn size of 54.17% TBSA. (5 g of SSD–Ag per 80 cm^2^); 20 days of medication.	Healing time for wounds treated with Acticoat was 12.42 ± 5.40 days, 3.35 days less than the control group (*p* < 0.01). At 15 days post-treatment, the healing percentage for the Acticoat group was 97.37%, higher than the control group but not significantly different. At the 6th day post-treatment, the bacterial clearance rate for the Acticoat group was 16.67% and, on the 12th day, it was 26.67%, both significantly higher than the control group, though no differences at the end of the study.	2007 [61]
Aquacel Ag and SSD	39 pediatric patients with partial-thickness burns treated with Aquacel Ag, 40 with SSD; the objective was to compare the hospital length of stay.	Patients treated with Aquacel Ag had a significantly shorter mean hospital stay (3.8 days) compared to those treated with SSD (5.9 days) (*p* = 0.001). Aquacel Ag adhered to the burn, reducing pain.	2007 [62]
Urgotul SSD vs. Contreet Ag	A retrospective cohort study was performed with 2 groups of 20 burns until wounds healed or grafted.	Pain was “absent or slight” in 61 (92%) dressing changes with Urgotul SSD and in 60 (85%) of the dressing changes with Contreet Ag. The dressing application was comparable. Contreet Ag had a greater ability to absorb exudate than Urgotul SSD.	2008 [63]
Silvasorb gel vs. Silvadene SSD	In a prospective, randomized study of 24 patients aged 2 months to 18 years, TBSA burns ranging from 1% to 40% were observed for 21 days or until full re-epithelialization.	SilvaSorb Gel was associated with significantly less pain compared to Silvadene, respectively (*p* = 0.004). No significant differences in the number of dressing changes (*p* = 0.383), re-epithelialization (*p* = 0.449), and rate of infection between the two dressings.	2009 [64]
Urgotul SSD (petroleum jelly with SSD) versus Silvadene SSD	68 patients with partial-thickness burn wounds less than 15%; monitored percentage of wound infection, total cost of wound dressing, pain medication, level of pain, and time of wound healing.	Time of wound closure was significantly shorter in the Urgotul SSD-treated group (10 ± 4 days bin Urgotul SSD- versus 12 ± 6 in 1% silver sulfadiazine-treated group) between both groups (*p* < 0.05). Average pain scores and pain medication in Urgotul SSD-treated group were significantly lower than the silver sulfadiazine-treated group (3 ± 1 versus 6 ± 2), *p* < 0.05.	2009 [65]
Aquacel Ag vs. 1% SSD	A prospective, randomized trial, 70 patients were equally divided, all with partial-thickness burns.	Time-to-wound closure was significantly shorter in the Aquacel^®^ Ag-treated group compared to the silver sulfadiazine-treated group (10 ± 3 days vs. 13.7 ± 4.3 days, *p* < 0.02). Number of hospital visits for dressing changes was significantly lower in the Aquacel^®^ Ag-treated group (3.5 ± 1 visits) compared to the silver sulfadiazine-treated group (13.7 ± 4 visits, *p* < 0.001). Average pain scores during dressing changes were significantly lower in the Aquacel^®^ Ag group than in the silver sulfadiazine group on days 1, 3, and 7. The scores were 4.1 ± 2.1, 2.1 ± 1.8, and 0.9 ± 1.4 for the Aquacel^®^ Ag group versus 6.1 ± 2.3, 5.2 ± 2.1, and 3.3 ± 1.9 for the silver sulfadiazine group, respectively (*p* < 0.02). Total cost of treatment was significantly lower for the Aquacel^®^ Ag group (52 ± 29 US dollars) compared to the silver sulfadiazine group (93 ± 36 US dollars, *p* < 0.01).	2010 [66]
Askina Calgitrol Ag (silver alginate/polyurethane foam) vs. SSD	65 patients with partial-thickness burn wounds, less than 24 h post-burn, with TBSA less than 15%; in the Askina Calgitrol Ag^®^ group (30), dressings were changed every 5 days, in the SSD group (35), dressings were changed daily.	Time to healing was significantly shorter in the Askina Calgitrol Ag^®^ group (7 ± 3.51 days) compared to the 1% Ag SD group (14 ± 4.18 days) (*p* < 0.02). Askina Calgitrol Ag^®^ group had significantly lower pain scores compared to the 1% SSD group (2.23 ± 1.87 vs. 6.08 ± 2.33) (*p* < 0.02). Nursing time was significantly reduced in the Askina Calgitrol Ag^®^ group (*p* < 0.02).	2010 [67]
Mepilex Ag vs. SSD	Open, parallel, randomized, comparative, multicenter study with patients, 5 years and older, with partial-thickness thermal burns (2.5–20% TBSA); a total of 101 patients.	Mean healing rates were 71.7% for the Mepilex Ag group and 60.8% for the SSD group. Mean time to discharge from inpatient hospital care was shorter for the Mepilex Ag group (5.62 days) compared to the SSD group (8.31 days) (*p* = 0.034), and no significant difference in average healing time was observed. Less pain upon application and during wear in the acute stages of wound healing with Mepilex Ag (statistically significant). More cost effective than SSD (data from subsamples of patients).	2011 [68]
Aquacel Ag vs. moist open burn ointment (MEBO)	40 patients with partial-thickness facial burns were equally divided between silver dressing and control.	Aquacel^®^ Ag group had a mean time of 10.5 days for reepithelization, compared to 12.4 days for the MEBO^®^ group (*p* < 0.05). Aquacel^®^ Ag group had softer, better-quality scars, though with some hyperpigmentation. Higher patient comfort was observed with Aquacel^®^ Ag.	2011 [69]
Aquacel Ag Burn Glove	Phase II non-comparative assessment of the management of partial thickness hand burns using a glove. 23 patients (mean age 41.2 years, male participants 74%) participated. The duration of treatment was 21 days.	A mean decrease in hand burn area from 29.4% at the baseline to 8.6% at the final evaluation, with 70% of hand burns fully re-epithelialized over 15.6 days. The mean pain score was 1.15 at rest and 2.29 during movement (0–10 range). Glove was well tolerated by patients.	2012 [70]
Aquacel Ag vs. SSD	Randomized trial of superficial partial-thickness burns, with 24 subjects, 18 men and 6 women, aged between 19 and 53 years.	The number of treatments required for 100% re-epithelialization was higher for the SSD group (10.27 ± 7.46) compared to the Aquacel Ag group (4.10 ± 1.38) (*p* = 0.02). SSD group reported a mean pain score of 4.70 ± 2.22, while the Aquacel Ag group reported a score of 2.92 ± 1.12. (*p* = 0.03).	2013 [71]
Aquacel^®^ Ag and Acticoat	A prospective, randomized, controlled study of 100 patients with partial-thickness burns.	No significant differences between the dressings in terms of wound healing and bacterial colonization (*p* = 0.226–0.941), Aquacel^®^ Ag had advantages regarding nurse experience (*p* < 0.001 to 0.125). Patients experienced similar baseline pain with both dressings. Reduced frequency of dressing changes in Aquacel group should be beneficial for the patient and nurse.	2014 [72]
Mepilex^™^ Ag vs. Acticoat^™^ and Acticoat^™^ + Mepitel^™^ Ag	Children aged 0–15 years with acute partial-thickness burns (superficial partial to deep partial thickness) and TBSA of ≤10%, a total of 103 participants.	Median days to 95% re-epithelialization were 9.50 days for Acticoat^™^, 10.00 days for Acticoat^™^ + Mepitel^™^, and 7.00 days for Mepilex Ag^TM^ (statistically significant) Mepilex Ag^™^ silicone dressings decreased the FLACC score (nurse’s observation of pain) by 37%, as compared to Acticoat^™^ (*p* = 0.002). Silicone-based dressings are useful for pediatric population since it reduces pain and wound trauma.	2015 [73]
Acticoat™ vs. Aquacel Ag	A single-blind, randomized controlled study in a Pediatric Emergency Department, included 89 children with superficial or mid-dermal burns (<10% TBSA), who were randomized to receive either the Acticoat™ (n = 45) or Aquacel^®^ Ag (n = 44) dressings.	No significant difference between the groups in terms of percentage epithelialization by day 10, with Acticoat™ showing 93 ± 14% and Aquacel^®^ Ag showing 94 ± 17% (*p* = 0.89). No significant difference in infection and escalation of care. Aquacel^®^ Ag dressings (59) required significantly fewer dressing changes compared to Acticoat (102) (*p* = 0.03)	2016 [74]
Procellera™ + Standard of Care (SoC) versus SoC (moleskin and Tegaderm)	A prospective randomized controlled two-arm Clinical Study for blister management. The study involved 80 Ranger recruits as participants in a 14-day study.	No significant difference in wound healing rates between the SoC group and the SoC + Procellera group (*p* = 0.528). No significant difference in pain management between the SoC and SoC + Procellera groups.	2017 [75]
Mepilex A vs. Suprathel (DL-polylactic acid membrane)	A prospective randomized controlled trial comparing the outpatient treatment of pediatric and adult partial-thickness burns. 29 adults and 33 pediatric patients (almost equally split between two dressings). TBSA: 1–29% in Meiplex Ag and 1–20% in Suprathel group.	The median time to complete reepithelialization was 12 days for both groups (*p* = 0.75). Suprathel reported better overall scar quality, and Mepilex Ag increased stiffness of burned skin at 1 month post-burn. Patients experienced less pain with Suprathel (only for first 5 days, *p* = 0.03).	2018 [76]
Silverlon vs. SSD or mafenide acetate (considered topical antimicrobials)	A 10-year retrospective analysis on a total of 987 combat burn casualties, with 184 patients in Group 1 (Silverlon) and 803 in Group 2 (topical antimicrobial); 49% of the cohort had third-degree burns.	The incidence of wound infection was 5.4% in Group 1 and 9.5% in Group 2 (*p* = 0.08), the overall mortality rate did not differ significantly between the groups (8% in Group 1). The incidence of bacteremia was 4.3% in Group 1 and 5.5% in Group 2, showing no significant difference (*p* = 1.0). Topical antimicrobials application was painful.	2018 [77]
Acticoat Flex 3 vs. 1% SSD	A randomized, single-center, single-blind trial involving 100 adults aged 18–65 with second-degree burns.	Reepithelization: Acticoat: 48% (24/50 patients), SSD: 52% (26/50 patients) (*p* = 0.56). Number of dressing changes: Acticoat fewer than SSD (*p* < 0.001)	2022 [78]
Procellera™ versus SoC (Standard of Care)	A single-center prospective, randomized controlled clinical trial with 38 patients with dermal burn/traumatic wounds. Procellera dressing compared with SOC: silver nylon, SSD ointment, bacitracin, xeroform, 5% sulfamylon solution, and Manuka honey, observations at 7-day.	In 52% of the Procellera-treated wounds, little to no biofilm could be detected by scanning electron microscopy compared to only 24% of SoC-treated wounds; Procellera lowered the increase in biofilm versus SoC (*p* < 0.05).	2024 [79]

## References

[1] Thaarup I.C., Iversen A.K.S., Lichtenberg M., Bjarnsholt T., Jakobsen T.H. (2022). Biofilm Survival Strategies in Chronic Wounds. Microorganisms.

[2] Järbrink K., Ni G., Sönnergren H., Schmidtchen A., Pang C., Bajpai R., Car J. (2016). Prevalence and Incidence of Chronic Wounds and Related Complications: A Protocol for a Systematic Review. Syst. Rev..

[3] Heyer K., Herberger K., Protz K., Glaeske G., Augustin M. (2016). Epidemiology of Chronic Wounds in Germany: Analysis of Statutory Health Insurance Data. Wound Repair Regen..

[4] Paladini F., Pollini M. (2019). Antimicrobial Silver Nanoparticles for Wound Healing Application: Progress and Future Trends. Materials.

[5] Guest J.F., Ayoub N., McIlwraith T., Uchegbu I., Gerrish A., Weidlich D., Vowden K., Vowden P. (2015). Health Economic Burden That Wounds Impose on the National Health Service in the UK. BMJ Open.

[6] Asaad A., Badr S. (2016). Surgical Site Infections in Developing Countries: Current Burden and Future Challenges. Clin. Microbiol. Open Access.

[7] Nussbaum S.R., Carter M.J., Fife C.E., DaVanzo J., Haught R., Nusgart M., Cartwright D. (2018). An Economic Evaluation of the Impact, Cost, and Medicare Policy Implications of Chronic Nonhealing Wounds. Value Health J. Int. Soc. Pharmacoecon. Outcomes Res..

[8] Highmore C.J., Melaugh G., Morris R.J., Parker J., Direito S.O.L., Romero M., Soukarieh F., Robertson S.N., Bamford N.C. (2022). Translational Challenges and Opportunities in Biofilm Science: A BRIEF for the Future. npj Biofilms Microbiomes.

[9] Wijesooriya L.I., Waidyathilake D. (2022). Antimicrobial Properties of Nonantibiotic Agents for Effective Treatment of Localized Wound Infections: A Minireview. Int. J. Low. Extrem. Wounds.

[10] Gould L.J., Alderden J., Aslam R., Barbul A., Bogie K.M., El Masry M., Graves L.Y., White-Chu E.F., Ahmed A., Boanca K. (2024). WHS Guidelines for the Treatment of Pressure Ulcers—2023 Update. Wound Repair Regen..

[11] Nímia H.H., Carvalho V.F., Isaac C., Souza F.Á., Gemperli R., Paggiaro A.O. (2019). Comparative Study of Silver Sulfadiazine with Other Materials for Healing and Infection Prevention in Burns: A Systematic Review and Meta-Analysis. Burns.

[12] Singh S., Young A., McNaught C.-E. (2017). The Physiology of Wound Healing. Surg. Oxf..

[13] Wang P.-H., Huang B.-S., Horng H.-C., Yeh C.-C., Chen Y.-J. (2018). Wound Healing. J. Chin. Med. Assoc. JCMA.

[14] Govindaraju P., Todd L., Shetye S., Monslow J., Puré E. (2019). CD44-Dependent Inflammation, Fibrogenesis, and Collagenolysis Regulates Extracellular Matrix Remodeling and Tensile Strength during Cutaneous Wound Healing. Matrix Biol..

[15] Goh T.C., Bajuri M.Y., Nadarajah S.C., Abdul Rashid A.H., Baharuddin S., Zamri K.S. (2020). Clinical and Bacteriological Profile of Diabetic Foot Infections in a Tertiary Care. J. Foot Ankle Res..

[16] Huang D., Wang J., Ren K., Ji J. (2020). Functionalized Biomaterials to Combat Biofilms. Biomater. Sci..

[17] Guzmán-Soto I., McTiernan C., Gonzalez-Gomez M., Ross A., Gupta K., Suuronen E.J., Mah T.-F., Griffith M., Alarcon E.I. (2021). Mimicking Biofilm Formation and Development: Recent Progress in in Vitro and in Vivo Biofilm Models. iScience.

[18] Bahamondez-Canas T.F., Heersema L.A., Smyth H.D.C. (2019). Current Status of In Vitro Models and Assays for Susceptibility Testing for Wound Biofilm Infections. Biomedicines.

[19] Darvishi S., Tavakoli S., Kharaziha M., Girault H.H., Kaminski C.F., Mela I. (2022). Advances in the Sensing and Treatment of Wound Biofilms. Angew. Chem. Int. Ed..

[20] Warriner R., Burrell R. (2005). Infection and the Chronic Wound: A Focus on Silver. Adv. Skin Wound Care.

[21] Serena T.E., Jalodi O., Serena L., Patel K., Mynti M. (2021). Evaluation of the Combination of a Biofilm-Disrupting Agent and Negative Pressure Wound Therapy: A Case Series. J. Wound Care.

[22] Wu J., Zhang F., Liu J., Yao H., Wang Y. (2023). Effect of Silver-Containing Hydrofiber Dressing on Burn Wound Healing: A Meta-Analysis and Systematic Review. J. Cosmet. Dermatol..

[23] Bourdillon K.A., Delury C.P., Cullen B.M. (2017). Biofilms and Delayed Healing—An in Vitro Evaluation of Silver- and Iodine-Containing Dressings and Their Effect on Bacterial and Human Cells. Int. Wound J..

[24] Carvalho C.D.S., Bernardes M.J.C., Gonçalves R.C., Vilela M.S., Silva M.V.M.D., Oliveira V.D.S., Rocha M.R.D., Vinaud M.C., Galdino H., Lino R.D.S. (2022). Treatment of Experimentally Induced Partial-Thickness Burns in Rats with Different Silver-Impregnated Dressings. Acta Cir. Bras..

[25] Kostenko V., Lyczak J., Turner K., Martinuzzi R.J. (2010). Impact of Silver-Containing Wound Dressings on Bacterial Biofilm Viability and Susceptibility to Antibiotics during Prolonged Treatment. Antimicrob. Agents Chemother..

[26] May A., Kopecki Z., Carney B., Cowin A. (2022). Antimicrobial Silver Dressings: A Review of Emerging Issues for Modern Wound Care. ANZ J. Surg..

[27] Metcalf D.G., Bowler P.G. (2020). Clinical Impact of an Anti-Biofilm Hydrofiber Dressing in Hard-to-Heal Wounds Previously Managed with Traditional Antimicrobial Products and Systemic Antibiotics. Burns Trauma.

[28] Doherty C., Byrne C.V., Baqader S., El-Chami C., McBain A.J., Thomason H.A. (2023). Anti-Biofilm Effects and Healing Promotion by Silver Oxynitrate-Based Dressings. Sci. Rep..

[29] Said J., Walker M., Parsons D., Stapleton P., Beezer A.E., Gaisford S. (2014). An in Vitro Test of the Efficacy of an Anti-Biofilm Wound Dressing. Int. J. Pharm..

[30] Nešporová K., Pavlík V., Šafránková B., Vágnerová H., Odráška P., Žídek O., Císařová N., Skoroplyas S., Kubala L., Velebný V. (2020). Effects of Wound Dressings Containing Silver on Skin and Immune Cells. Sci. Rep..

[31] Bowler P.G., Parsons D. (2016). Combatting Wound Biofilm and Recalcitrance with a Novel Anti-Biofilm Hydrofiber^®^ Wound Dressing. Wound Med..

[32] Seth A.K., Zhong A., Nguyen K.T., Hong S.J., Leung K.P., Galiano R.D., Mustoe T.A. (2014). Impact of a Novel, Antimicrobial Dressing on in Vivo, *Pseudomonas aeruginosa* Wound Biofilm: Quantitative Comparative Analysis Using a Rabbit Ear Model. Wound Repair Regen..

[33] Regulski M., Myntti M.F., James G.A. (2023). Anti-Biofilm Efficacy of Commonly Used Wound Care Products in In Vitro Settings. Antibiotics.

[34] Davis S.C., Gil J., Solis M., Higa A., Mills A., Simms C., Pena P.V., Li J., Raut V. (2022). Antimicrobial Effectiveness of Wound Matrices Containing Native Extracellular Matrix with Polyhexamethylene Biguanide. Int. Wound J..

[35] Suleman L., Purcell L., Thomas H., Westgate S. (2020). Use of Internally Validated in Vitro Biofilm Models to Assess Antibiofilm Performance of Silver-Containing Gelling Fibre Dressings. J. Wound Care.

[36] Kim H., Makin I., Skiba J., Ho A., Housler G., Stojadinovic A., Izadjoo M. (2014). Antibacterial Efficacy Testing of a Bioelectric Wound Dressing Against Clinical Wound Pathogens. Open Microbiol. J..

[37] White R., Cowan T., Glover D. (2011). Evidence-Based Dressing Selection. J. Wound Care.

[38] Karlsmark T., Agerslev R.H., Bendz S.H., Larsen J.R., Roed-Petersen J., Andersen K.E. (2003). Clinical Performance of a New Silver Dressing, Contreet Foam, for Chronic Exuding Venous Leg Ulcers. J. Wound Care.

[39] Jørgensen B., Price P., Andersen K.E., Gottrup F., Bech-Thomsen N., Scanlon E., Kirsner R., Rheinen H., Roed-Petersen J., Romanelli M. (2005). The Silver-Releasing Foam Dressing, Contreet Foam, Promotes Faster Healing of Critically Colonised Venous Leg Ulcers: A Randomised, Controlled Trial. Int. Wound J..

[40] Meaume S., Vallet D., Morere M.N., Téot L. (2005). Evaluation of a Silver-Releasing Hydroalginate Dressing in Chronic Wounds with Signs of Local Infection. J. Wound Care.

[41] Münter K.C., Beele H., Russell L., Crespi A., Gröchenig E., Basse P., Alikadic N., Fraulin F., Dahl C., Jemma A.P. (2006). Effect of a Sustained Silver-Releasing Dressing on Ulcers with Delayed Healing: The CONTOP Study. J. Wound Care.

[42] Jude E.B., Apelqvist J., Spraul M., Martini J., Silver Dressing Study Group (2007). Prospective Randomized Controlled Study of Hydrofiber Dressing Containing Ionic Silver or Calcium Alginate Dressings in Non-Ischaemic Diabetic Foot Ulcers. Diabet. Med. J. Br. Diabet. Assoc..

[43] Lazareth I., Meaume S., Sigal-Grinberg M.L., Combemale P., Guyadec T.L., Zagnoli A., Perrot J.-L., Sauvadet A., Bohbot S. (2008). The Role of a Silver Releasing Lipido-Colloid Contact Layer in Venous Leg Ulcers Presenting Inflammatory Signs Suggesting Heavy Bacterial Colonization: Results of a Randomized Controlled Study. Wounds Compend. Clin. Res. Pract..

[44] Gago M., Garcia F., Gaztelu V., Verdu J., Lopez P., Nolasco A. (2008). A Comparison of Three Silver-Containing Dressings in the Treatment of Infected, Chronic Wounds. Wounds Compend. Clin. Res. Pract..

[45] Michaels J.A., Campbell B., King B., Palfreyman S.J., Shackley P., Stevenson M. (2009). Randomized Controlled Trial and Cost-Effectiveness Analysis of Silver-Donating Antimicrobial Dressings for Venous Leg Ulcers (VULCAN Trial). Br. J. Surg..

[46] Miller C.N., Newall N., Kapp S.E., Lewin G., Karimi L., Carville K., Gliddon T., Santamaria N.M. (2010). A Randomized-Controlled Trial Comparing Cadexomer Iodine and Nanocrystalline Silver on the Healing of Leg Ulcers. Wound Repair Regen..

[47] Chuangsuwanich A., Charnsanti O., Lohsiriwat V., Kangwanpoom C., Thong-In N. (2011). The Efficacy of Silver Mesh Dressing Compared with Silver Sulfadiazine Cream for the Treatment of Pressure Ulcers. J. Med. Assoc. Thail. Chotmaihet Thangphaet.

[48] Lazareth I., Meaume S., Sigal-Grinberg M.L., Combemale P., Le Guyadec T., Zagnoli A. (2012). Efficacy of a Silver Lipidocolloid Dressing on Heavily Colonised Wounds: A Republished RCT. J. Wound Care.

[49] Harding K., Gottrup F., Jawień A., Mikosiński J., Twardowska-Saucha K., Kaczmarek S., Sopata M., Shearman C., Pieronne A., Kommala D. (2012). A Prospective, Multi-Centre, Randomised, Open Label, Parallel, Comparative Study to Evaluate Effects of AQUACEL^®^ Ag and Urgotul^®^ Silver Dressing on Healing of Chronic Venous Leg Ulcers. Int. Wound J..

[50] Senet P., Bause R., Jørgensen B., Fogh K. (2014). Clinical Efficacy of a Silver-Releasing Foam Dressing in Venous Leg Ulcer Healing: A Randomised Controlled Trial. Int. Wound J..

[51] Brouillard C., Bursztejn A.-C., Latarche C., Cuny J.-F., Truchetet F., Goullé J.-P., Schmutz J.-L. (2018). Silver Absorption and Toxicity Evaluation of Silver Wound Dressings in 40 Patients with Chronic Wounds. J. Eur. Acad. Dermatol. Venereol. JEADV.

[52] Hurd T., Woodmansey E.J., Watkins H.M.A. (2021). A Retrospective Review of the Use of a Nanocrystalline Silver Dressing in the Management of Open Chronic Wounds in the Community. Int. Wound J..

[53] Wang Y.-C., Lee H.-C., Chen C.-L., Kuo M.-C., Ramachandran S., Chen R.-F., Kuo Y.-R. (2021). The Effects of Silver-Releasing Foam Dressings on Diabetic Foot Ulcer Healing. J. Clin. Med..

[54] Wang R., Guo Y., Li B., Zheng J., Tang Z., Shu M. (2022). Application Effect of Silver-Containing Dressings in the Repair of Chronic Refractory Wounds. Evid.-Based Complement. Altern. Med. ECAM.

[55] Dissemond J., Aare K., Ozer K., Gandhi D., Ryan J.L., DeKoven M. (2023). Aquacel Ag Advantage/Ag+ Extra and Cutimed Sorbact in the Management of Hard-to-Heal Wounds: A Cohort Study. J. Wound Care.

[56] Lafontaine N., Jolley J., Kyi M., King S., Iacobaccio L., Staunton E., Wilson B., Seymour C., Rogasch S., Wraight P. (2023). Prospective Randomised Placebo-Controlled Trial Assessing the Efficacy of Silver Dressings to Enhance Healing of Acute Diabetes-Related Foot Ulcers. Diabetologia.

[57] Tredget E.E., Shankowsky H.A., Groeneveld A., Burrell R. (1998). A Matched-Pair, Randomized Study Evaluating the Efficacy and Safety of Acticoat Silver-Coated Dressing for the Treatment of Burn Wounds. J. Burn Care Rehabil..

[58] Caruso D.M., Foster K.N., Hermans M.H.E., Rick C. (2004). Aquacel Ag in the Management of Partial-Thickness Burns: Results of a Clinical Trial. J. Burn Care Rehabil..

[59] Varas R.P., O’Keeffe T., Namias N., Pizano L.R., Quintana O.D., Herrero Tellachea M., Rashid Q., Ward C.G. (2005). A Prospective, Randomized Trial of Acticoat versus Silver Sulfadiazine in the Treatment of Partial-Thickness Burns: Which Method Is Less Painful?. J. Burn Care Rehabil..

[60] Caruso D.M., Foster K.N., Blome-Eberwein S.A., Twomey J.A., Herndon D.N., Luterman A., Silverstein P., Antimarino J.R., Bauer G.J. (2006). Randomized Clinical Study of Hydrofiber Dressing with Silver or Silver Sulfadiazine in the Management of Partial-Thickness Burns. J. Burn Care Res..

[61] Huang Y., Li X., Liao Z., Zhang G., Liu Q., Tang J., Peng Y., Liu X., Luo Q. (2007). A Randomized Comparative Trial between Acticoat and SD-Ag in the Treatment of Residual Burn Wounds, Including Safety Analysis. Burns.

[62] Paddock H.N., Fabia R., Giles S., Hayes J., Lowell W., Besner G.E. (2007). A Silver Impregnated Antimicrobial Dressing Reduces Hospital Length of Stay for Pediatric Patients with Burns. J. Burn Care Res..

[63] Jester I., Bohn I., Hannmann T., Waag K.-L., Loff S. (2008). Comparison of Two Silver Dressings for Wound Management in Pediatric Burns. Wounds Compend. Clin. Res. Pract..

[64] Glat P.M., Kubat W.D., Hsu J.F., Copty T., Burkey B.A., Davis W., Goodwin I. (2009). Randomized Clinical Study of SilvaSorb Gel in Comparison to Silvadene Silver Sulfadiazine Cream in the Management of Partial-Thickness Burns. J. Burn Care Res..

[65] Muangman P., Muangman S., Opasanon S., Keorochana K., Chuntrasakul C. (2009). Benefit of Hydrocolloid SSD Dressing in the Outpatient Management of Partial Thickness Burns. J. Med. Assoc. Thail. Chotmaihet Thangphaet.

[66] Muangman P., Pundee C., Opasanon S. (2010). A Prospective, Randomized Trial of Silver Containing Hydrofiber Dressing versus 1% Silver Sulfadiazine for the Treatment of Partial Thickness Burns. Int. Wound J..

[67] Opasanon S., Muangman P., Namviriyachote N. (2010). Clinical Effectiveness of Alginate Silver Dressing in Outpatient Management of Partial-Thickness Burns. Int. Wound J..

[68] Silverstein P., Heimbach D., Meites H., Latenser B., Mozingo D., Mullins F., Garner W., Turkowski J., Shupp J., Glat P. (2011). An Open, Parallel, Randomized, Comparative, Multicenter Study to Evaluate the Cost-Effectiveness, Performance, Tolerance, and Safety of a Silver-Containing Soft Silicone Foam Dressing (Intervention) vs Silver Sulfadiazine Cream. J. Burn Care Res..

[69] Mabrouk A., Boughdadi N., Helal H., Zaki B., Maher A. (2011). Moist Occlusive Dressing (Aquacel^®^ Ag) versus Moist Open Dressing (MEBO^®^) in the Management of Partial-Thickness Facial Burns: A Comparative Study in Ain Shams University. Burns.

[70] Duteille F., Jeffery S.L.A. (2012). A Phase II Prospective, Non-Comparative Assessment of a New Silver Sodium Carboxymethylcellulose (AQUACEL^®^ Ag BURN) Glove in the Management of Partial Thickness Hand Burns. Burns.

[71] Yarboro D.D. (2013). A Comparative Study of the Dressings Silver Sulfadiazine and Aquacel Ag in the Management of Superficial Partial-Thickness Burns. Adv. Skin Wound Care.

[72] Verbelen J., Hoeksema H., Heyneman A., Pirayesh A., Monstrey S. (2014). Aquacel^®^ Ag Dressing versus Acticoat^TM^ Dressing in Partial Thickness Burns: A Prospective, Randomized, Controlled Study in 100 Patients. Part 1: Burn Wound Healing. Burns.

[73] Gee Kee E.L., Kimble R.M., Cuttle L., Khan A., Stockton K.A. (2015). Randomized Controlled Trial of Three Burns Dressings for Partial Thickness Burns in Children. Burns.

[74] Brown M., Dalziel S.R., Herd E., Johnson K., Wong She R., Shepherd M. (2016). A Randomized Controlled Study of Silver-Based Burns Dressing in a Pediatric Emergency Department. J. Burn Care Res..

[75] Housler G.J., Cross S., Marcel V., Kennedy D.O., Husband M., Register A., Roberts T., Grubbs S., Dudewicz D., Setka N. (2017). A Prospective Randomized Controlled Two-Arm Clinical Study Evaluating the Efficacy of a Bioelectric Dressing System for Blister Management in US Army Ranger Recruits. J. Spec. Oper. Med..

[76] Hundeshagen G., Collins V.N., Wurzer P., Sherman W., Nunez Lopez O., Sheaffer J., Herndon D.N., Finnerty C.C., Branski L.K. (2018). A Prospective, Randomized, Controlled Trial Comparing the Outpatient Treatment of Pediatric and Adult Partial-Thickness Burns with Suprathel or Mepilex Ag. J. Burn Care Res..

[77] Aurora A., Beasy A., Rizzo J.A., Chung K.K. (2018). The Use of a Silver-Nylon Dressing During Evacuation of Military Burn Casualties. J. Burn Care Res..

[78] Moreira S.S., de Camargo M.C., Caetano R., Alves M.R., Itria A., Pereira T.V., Lopes L.C. (2022). Efficacy and Costs of Nanocrystalline Silver Dressings versus 1% Silver Sulfadiazine Dressings to Treat Burns in Adults in the Outpatient Setting: A Randomized Clinical Trial. Burns.

[79] Chan R.K., Nuutila K., Mathew-Steiner S.S., Diaz V., Anselmo K., Batchinsky M., Carlsson A., Ghosh N., Sen C.K., Roy S. (2024). A Prospective, Randomized, Controlled Study to Evaluate the Effectiveness of a Fabric-Based Wireless Electroceutical Dressing Compared to Standard-of-Care Treatment Against Acute Trauma and Burn Wound Biofilm Infection. Adv. Wound Care.

[80] Sen C.K., Roy S., Mathew-Steiner S.S., Gordillo G.M. (2021). Biofilm Management in Wound Care. Plast. Reconstr. Surg..

[81] Geesey G.G., Richardson W.T., Yeomans H.G., Irvin R.T., Costerton J.W. (1977). Microscopic Examination of Natural Sessile Bacterial Populations from an Alpine Stream. Can. J. Microbiol..

[82] Costerton J.W., Geesey G.G., Cheng K.J. (1978). How Bacteria Stick. Sci. Am..

[83] Cámara M., Green W., MacPhee C.E., Rakowska P.D., Raval R., Richardson M.C., Slater-Jefferies J., Steventon K., Webb J.S. (2022). Economic Significance of Biofilms: A Multidisciplinary and Cross-Sectoral Challenge. npj Biofilms Microbiomes.

[84] Percival S.L., Hill K.E., Malic S., Thomas D.W., Williams D.W. (2011). Antimicrobial Tolerance and the Significance of Persister Cells in Recalcitrant Chronic Wound Biofilms. Wound Repair Regen..

[85] Fux C.A., Costerton J.W., Stewart P.S., Stoodley P. (2005). Survival Strategies of Infectious Biofilms. Trends Microbiol..

[86] Keren I., Kaldalu N., Spoering A., Wang Y., Lewis K. (2004). Persister Cells and Tolerance to Antimicrobials. FEMS Microbiol. Lett..

[87] Han G., Ceilley R. (2017). Chronic Wound Healing: A Review of Current Management and Treatments. Adv. Ther..

[88] Bessa L.J., Fazii P., Di Giulio M., Cellini L. (2015). Bacterial Isolates from Infected Wounds and Their Antibiotic Susceptibility Pattern: Some Remarks about Wound Infection. Int. Wound J..

[89] Kirketerp-Møller K., Jensen P.Ø., Fazli M., Madsen K.G., Pedersen J., Moser C., Tolker-Nielsen T., Høiby N., Givskov M., Bjarnsholt T. (2008). Distribution, Organization, and Ecology of Bacteria in Chronic Wounds. J. Clin. Microbiol..

[90] Fazli M., Bjarnsholt T., Kirketerp-Møller K., Jørgensen B., Andersen A.S., Krogfelt K.A., Givskov M., Tolker-Nielsen T. (2009). Nonrandom Distribution of *Pseudomonas aeruginosa* and *Staphylococcus aureus* in Chronic Wounds. J. Clin. Microbiol..

[91] Metcalf D.G., Bowler P.G. (2015). Biofilm Delays Wound Healing: A Review of the Evidence. Burns Trauma.

[92] James G.A., Swogger E., Wolcott R., Pulcini E.d., Secor P., Sestrich J., Costerton J.W., Stewart P.S. (2008). Biofilms in Chronic Wounds. Wound Repair Regen..

[93] Posnett J., Franks P.J. (2008). The Burden of Chronic Wounds in the UK. Nurs. Times.

[94] Harding K., Posnett J., Vowden K. (2013). A New Methodology for Costing Wound Care. Int. Wound J..

[95] Wolcott R.D., Rhoads D.D., Dowd S.E. (2008). Biofilms and Chronic Wound Inflammation. J. Wound Care.

[96] White R.J., Cutting K.F. (2012). Wound Biofilms-Are They Visible?. J. Wound Care.

[97] Versey Z., da Cruz Nizer W.S., Russell E., Zigic S., DeZeeuw K.G., Marek J.E., Overhage J., Cassol E. (2021). Biofilm-Innate Immune Interface: Contribution to Chronic Wound Formation. Front. Immunol..

[98] James G.A., Ge Zhao A., Usui M., Underwood R.A., Nguyen H., Beyenal H., deLancey Pulcini E., Agostinho Hunt A., Bernstein H.C., Fleckman P. (2016). Microsensor and Transcriptomic Signatures of Oxygen Depletion in Biofilms Associated with Chronic Wounds. Wound Repair Regen..

[99] Malone M., Swanson T. (2017). Biofilm-Based Wound Care: The Importance of Debridement in Biofilm Treatment Strategies. Br. J. Community Nurs..

[100] Nusbaum A.G., Gil J., Rippy M.K., Warne B., Valdes J., Claro A., Davis S.C. (2012). Effective Method to Remove Wound Bacteria: Comparison of Various Debridement Modalities in an in Vivo Porcine Model. J. Surg. Res..

[101] Sharma A., Sharma D., Zhao F. (2023). Updates on Recent Clinical Assessment of Commercial Chronic Wound Care Products. Adv. Healthc. Mater..

[102] Simões D., Miguel S.P., Ribeiro M.P., Coutinho P., Mendonça A.G., Correia I.J. (2018). Recent Advances on Antimicrobial Wound Dressing: A Review. Eur. J. Pharm. Biopharm..

[103] Dutta P.K., Wang B., Shrestha S. (2021). Silver in Health and Medicinal Applications, Amazon Kindle e-book ed.: Seattle, WA, USA. https://a.co/d/cGPPwNl.

[104] Dakal T.C., Kumar A., Majumdar R.S., Yadav V. (2016). Mechanistic Basis of Antimicrobial Actions of Silver Nanoparticles. Front. Microbiol..

[105] Rybka M., Mazurek Ł., Konop M. (2022). Beneficial Effect of Wound Dressings Containing Silver and Silver Nanoparticles in Wound Healing-From Experimental Studies to Clinical Practice. Life.

[106] Bedlovičová Z., Strapáč I., Baláž M., Salayová A. (2020). A Brief Overview on Antioxidant Activity Determination of Silver Nanoparticles. Molecules.

[107] Sullivan T.P., Eaglstein W.H., Davis S.C., Mertz P. (2001). The Pig as a Model for Human Wound Healing. Wound Repair Regen..

[108] Meyer W., Görgen S., Schlesinger C. (1986). Structural and Histochemical Aspects of Epidermis Development of Fetal Porcine Skin. Am. J. Anat..

[109] Roy S., Biswas S., Khanna S., Gordillo G., Bergdall V., Green J., Marsh C.B., Gould L.J., Sen C.K. (2009). Characterization of a Preclinical Model of Chronic Ischemic Wound. Physiol. Genomics.

[110] Patil P., Martin J.R., Sarett S.M., Pollins A.C., Cardwell N.L., Davidson J.M., Guelcher S.A., Nanney L.B., Duvall C.L. (2017). Porcine Ischemic Wound-Healing Model for Preclinical Testing of Degradable Biomaterials. Tissue Eng. Part C Methods.

[111] Trejo-Hernández A., Andrade-Domínguez A., Hernández M., Encarnación S. (2014). Interspecies Competition Triggers Virulence and Mutability in *Candida Albicans*—*Pseudomonas Aeruginosa* Mixed Biofilms. ISME J..

[112] Lansdown A.B.G., Williams A., Chandler S., Benfield S. (2005). Silver Absorption and Antibacterial Efficacy of Silver Dressings. J. Wound Care.

[113] Thuptimdang P., Limpiyakorn T., McEvoy J., Prüß B.M., Khan E. (2015). Effect of Silver Nanoparticles on *Pseudomonas Putida* Biofilms at Different Stages of Maturity. J. Hazard. Mater..

[114] Jensen P.Ø., Bjarnsholt T., Phipps R., Rasmussen T.B., Calum H., Christoffersen L., Moser C., Williams P., Pressler T., Givskov M. (2007). Rapid Necrotic Killing of Polymorphonuclear Leukocytes Is Caused by Quorum-Sensing-Controlled Production of Rhamnolipid by *Pseudomonas aeruginosa*. Microbiol. Read. Engl..

[115] Pearson J.P., Pesci E.C., Iglewski B.H. (1997). Roles of Pseudomonas Aeruginosa Las and Rhl Quorum-Sensing Systems in Control of Elastase and Rhamnolipid Biosynthesis Genes. J. Bacteriol..

[116] Miller K.G., Tran P.L., Haley C.L., Kruzek C., Colmer-Hamood J.A., Myntti M., Hamood A.N. (2014). Next Science Wound Gel Technology, a Novel Agent That Inhibits Biofilm Development by Gram-Positive and Gram-Negative Wound Pathogens. Antimicrob. Agents Chemother..

[117] Fox C.L. (1968). Silver Sulfadiazine—A New Topical Therapy for Pseudomonas in Burns: Therapy of *Pseudomonas* Infection in Burns. Arch. Surg..

[118] Percival S.L., Mayer D., Salisbury A.-M. (2017). Efficacy of a Surfactant-Based Wound Dressing on Biofilm Control. Wound Repair Regen..

[119] Ahmadi M., Adibhesami M. (2017). The Effect of Silver Nanoparticles on Wounds Contaminated with *Pseudomonas aeruginosa* in Mice: An Experimental Study. Iran. J. Pharm. Res. IJPR.

[120] Chaudhari P.R., Masurkar S.A., Shidore V.B., Kamble S.P. (2012). Effect of Biosynthesized Silver Nanoparticles on *Staphylococcus aureus* Biofilm Quenching and Prevention of Biofilm Formation. Nano-Micro Lett..

[121] Habash M.B., Park A.J., Vis E.C., Harris R.J., Khursigara C.M. (2014). Synergy of Silver Nanoparticles and Aztreonam against *Pseudomonas aeruginosa* PAO1 Biofilms. Antimicrob. Agents Chemother..

[122] Khansa I., Schoenbrunner A.R., Kraft C.T., Janis J.E. (2019). Silver in Wound Care-Friend or Foe?: A Comprehensive Review. Plast. Reconstr. Surg. Glob. Open.

[123] Poon V.K.M., Burd A. (2004). In Vitro Cytotoxity of Silver: Implication for Clinical Wound Care. Burns.

[124] Maghsoudi H., Monshizadeh S., Mesgari M. (2011). A Comparative Study of the Burn Wound Healing Properties of Saline-Soaked Dressing and Silver Sulfadiazine in Rats. Indian J. Surg..

[125] Cho Lee A.-R., Leem H., Lee J., Park K.C. (2005). Reversal of Silver Sulfadiazine-Impaired Wound Healing by Epidermal Growth Factor. Biomaterials.

[126] Rosen J., Landriscina A., Kutner A., Adler B.L., Krausz A.E., Nosanchuk J.D., Friedman A.J. (2015). Silver Sulfadiazine Retards Wound Healing in Mice via Alterations in Cytokine Expression. J. Investig. Dermatol..

[127] Qian L.-W., Fourcaudot A.B., Leung K.P. (2017). Silver Sulfadiazine Retards Wound Healing and Increases Hypertrophic Scarring in a Rabbit Ear Excisional Wound Model. J. Burn Care Res..

[128] Burd A., Kwok C.H., Hung S.C., Chan H.S., Gu H., Lam W.K., Huang L. (2007). A Comparative Study of the Cytotoxicity of Silver-Based Dressings in Monolayer Cell, Tissue Explant, and Animal Models. Wound Repair Regen..

[129] Capella-Monsonís H., Tilbury M.A., Wall J.G., Zeugolis D.I. (2020). Porcine Mesothelium Matrix as a Biomaterial for Wound Healing Applications. Mater. Today Bio.

[130] Dissemond J., Böttrich J.G., Braunwarth H., Hilt J., Wilken P., Münter K.-C. (2017). Evidence for Silver in Wound Care—Meta-Analysis of Clinical Studies from 2000–2015. JDDG J. Dtsch. Dermatol. Ges..

[131] Vermeulen H., van Hattem J.M., Storm-Versloot M.N., Ubbink D.T. (2007). Topical Silver for Treating Infected Wounds. Cochrane Database Syst. Rev..

[132] Storm-Versloot M.N., Vos C.G., Ubbink D.T., Vermeulen H. (2010). Topical Silver for Preventing Wound Infection. Cochrane Database Syst. Rev..

[133] Schaper N.C., van Netten J.J., Apelqvist J., Bus S.A., Hinchliffe R.J., Lipsky B.A., IWGDF Editorial Board (2020). Practical Guidelines on the Prevention and Management of Diabetic Foot Disease (IWGDF 2019 Update). Diabetes Metab. Res. Rev..

[134] Leaper D. (2012). Appropriate Use of Silver Dressings in Wounds: International Consensus Document. Int. Wound J..

